# Supplementary material for: Vaccination strategies to achieve outbreak control for MPXV Clade I with a one-time mass campaign in sub-Saharan Africa: A scenario-based modelling study
Source: PLoS Med. 2025 Sep 5;22(9):e1004726. doi: 10.1371/journal.pmed.1004726 (PMC12412972; doi:10.1371/journal.pmed.1004726)
Supplement: S2 Text — This document includes six sensitivity analyses conducted to evaluate the model assumptions. (S2_Text.DOCX) [file pmed.1004726.s002.docx]

**Supplementary information—S2 Text: Sensitivity analyses**

[Model re-calibration using all components of the synthetic contact matrices 2](#_Toc206682577)

[Waning immunity from smallpox vaccines 7](#_Toc206682578)

[Higher smallpox vaccine coverage in sub-Saharan African countries 11](#_Toc206682579)

[Scaling the next generation matrix with uncertainty adjustments 15](#_Toc206682580)

[Alternative maximum uptake threshold of 70% 19](#_Toc206682581)

[Alternative vaccine effectiveness of 75% or 85% 22](#_Toc206682582)

[References 27](#_Toc206682583)

**Model re-calibration using all components of the synthetic contact matrices**

To account for the potential presence of close contacts in non-household settings (e.g., schools), despite limited supporting evidence as of May 2025, we conducted a sensitivity analysis in which the model was re-calibrated using all components of the synthetic contact matrices [1]. As suggested in the preliminary study [2], this approach resulted in worse model fits compared to calibration using home contact components only (Figs A and A in S1 Text), with lower likelihood values and higher Akaike Information Criterion (AIC) scores.


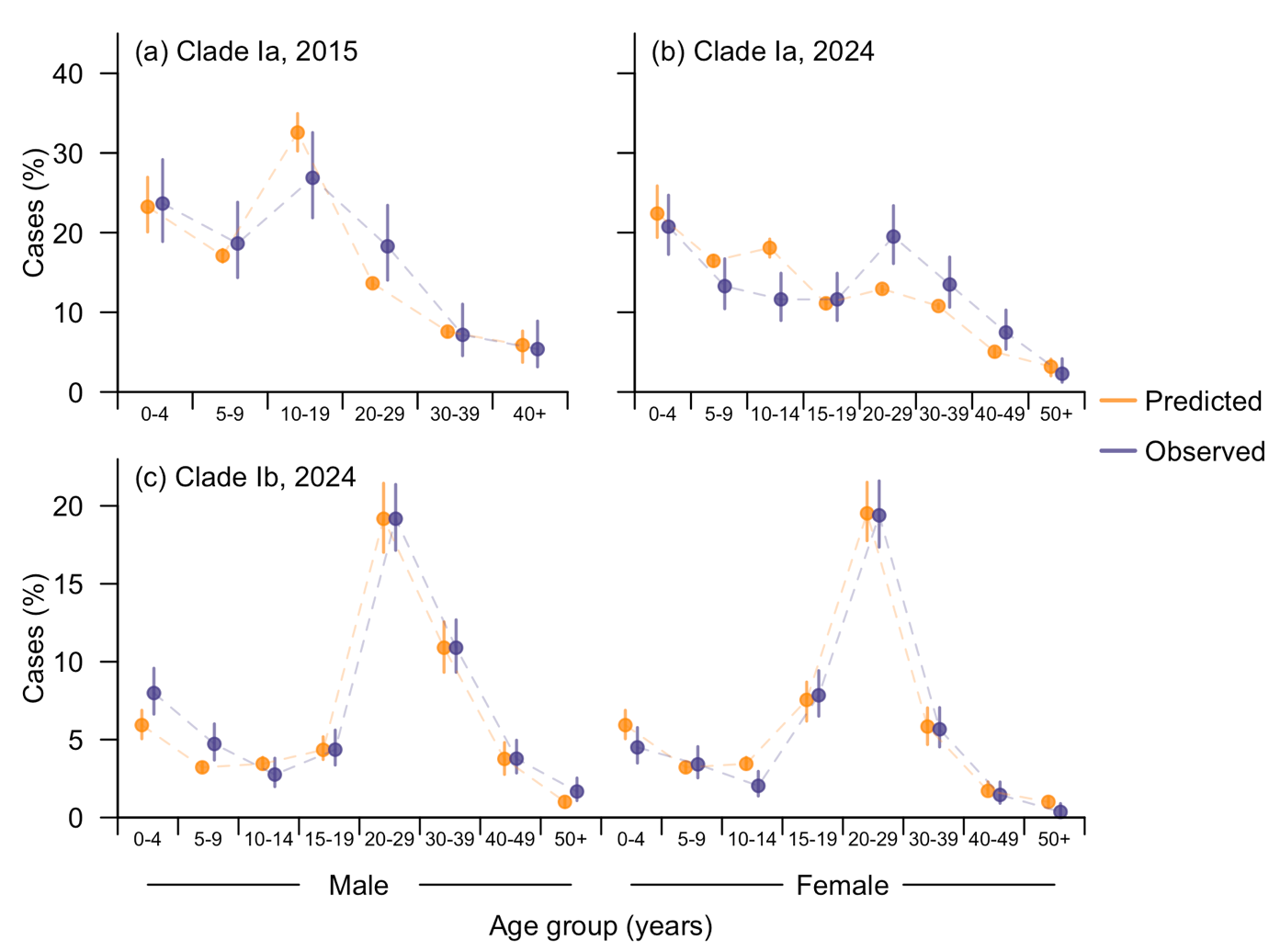


**Fig A.** Fitted case demographics for the DRC, based on the model calibrated using all contact components of the synthetic contact matrices [1]. Subfigure (a) and (b) show the age distribution of case counts in endemic provinces in the DRC for the years 2015 and 2024, respectively, while subfigure (c) presents the age and sex distribution of cases counts in South Kivu, the DRC in 2024. Observed case demographics were obtained from Murayama et al [2].

The projected $R_{eff}$ values for the 47 sub-Saharan African countries were generally lower compared to those in the main analysis. For instance, in the DRC in 2024, the $R_{eff}$ for Clade Ia transmission was estimated at 0.85 (95% CI: 0.85–0.86), while that for Clade Ib transmission was 1.44 (95% CI: 1.36–1.51), with additional sexual transmission accounting for 43.6% (95% CI: 40.5%–46.6%) to overall transmission. This led to substantially lower projected mpox vaccine demands across all the 47 countries and under all four scenarios with varying levels of sexual transmission (Figs B–E). Nevertheless, the age prioritisation for vaccination in the case of limited vaccine supply was similar to the main analysis.


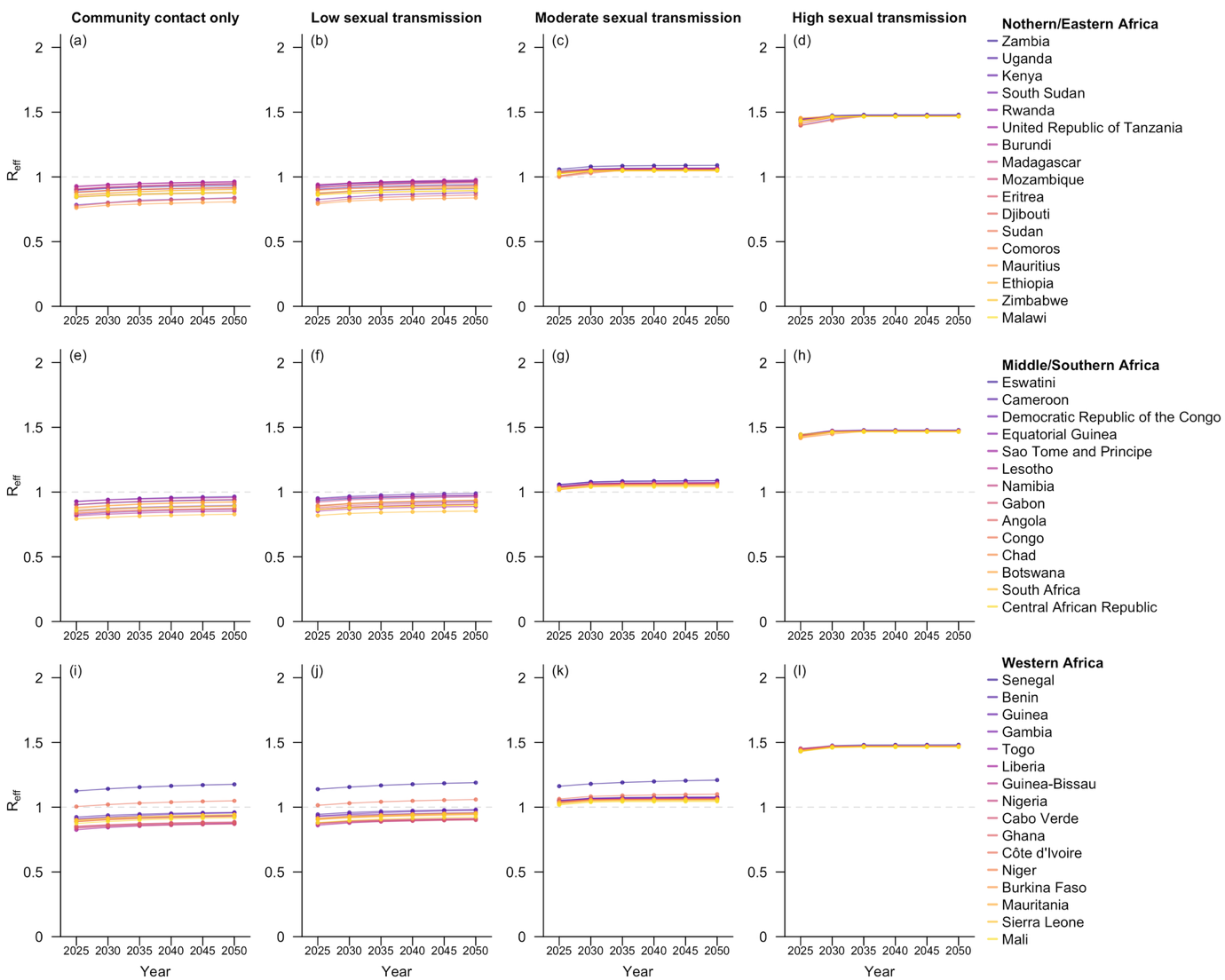


**Fig B** Projected $R_{eff}$ for the 47 sub-Saharan African countries at five-year intervals from 2025 to 2050, based on the model calibrated using all contact components of the synthetic contact matrices [1]. Countries within the same geospatial subregions are grouped in the same row and arranged in descending order by their estimated $R_{eff}$ in 2050. The four columns correspond to the four hypothetical scenarios with varying levels of sexual transmission.

**
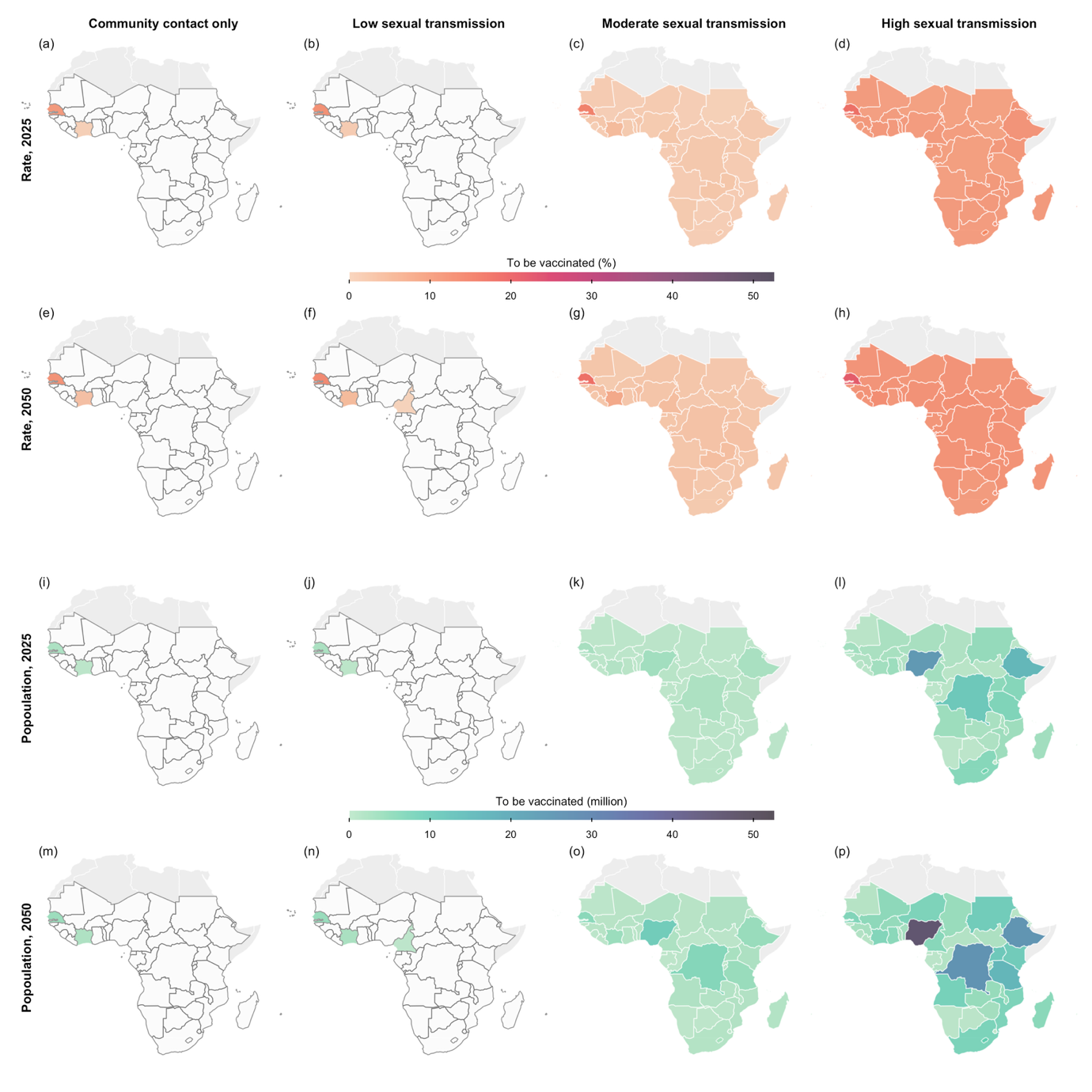
**

**Fig C.** Projected minimal vaccine demand, based on the model calibrated using all contact components of the synthetic contact matrices [1]. Shown are the vaccine coverage rates (Row 1 and 2) and the number of individuals requiring vaccination (Row 3 and 4) to prevent secondary infections in each sub-Saharan African country modelled for 2025 (Row 1 and 3) and 2050 (Row 2 and 4), assuming mass vaccination. The four columns correspond to the four hypothetical scenarios with varying levels of sexual transmission. Countries which do not require vaccination are coloured in white, with borders outlined in dark grey. The base map layer (boundaries of African countries) is sourced from Natural Earth (https://www.naturalearthdata.com), available under the Public Domain license (https://www.naturalearthdata.com/about/terms-of-use/).


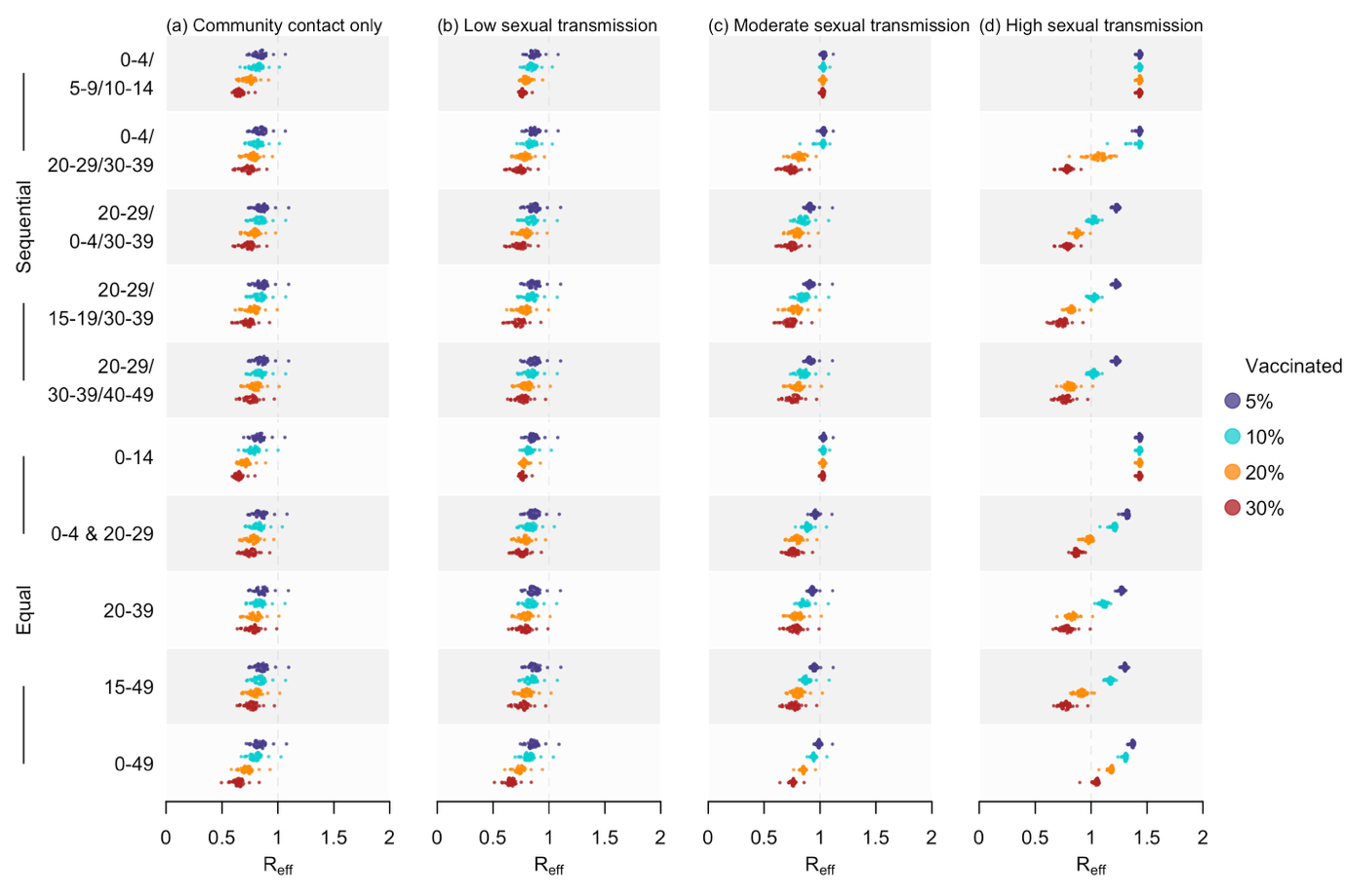


**Fig D**. Estimated $R_{eff}$ in 2025 under diverse vaccination strategies, based on the model calibrated using all contact components of the synthetic contact matrices [1]. Doses were allocated using two allocation methods, including sequential (first five rows) and equal (last five rows). Four coverage rates, including 5%, 10%, 20%, and 30%, were assessed, with outcomes summarised as shaded distributions in purple, blue, orange, and red, respectively. The four columns correspond to the four hypothetical scenarios with varying levels of sexual transmission.


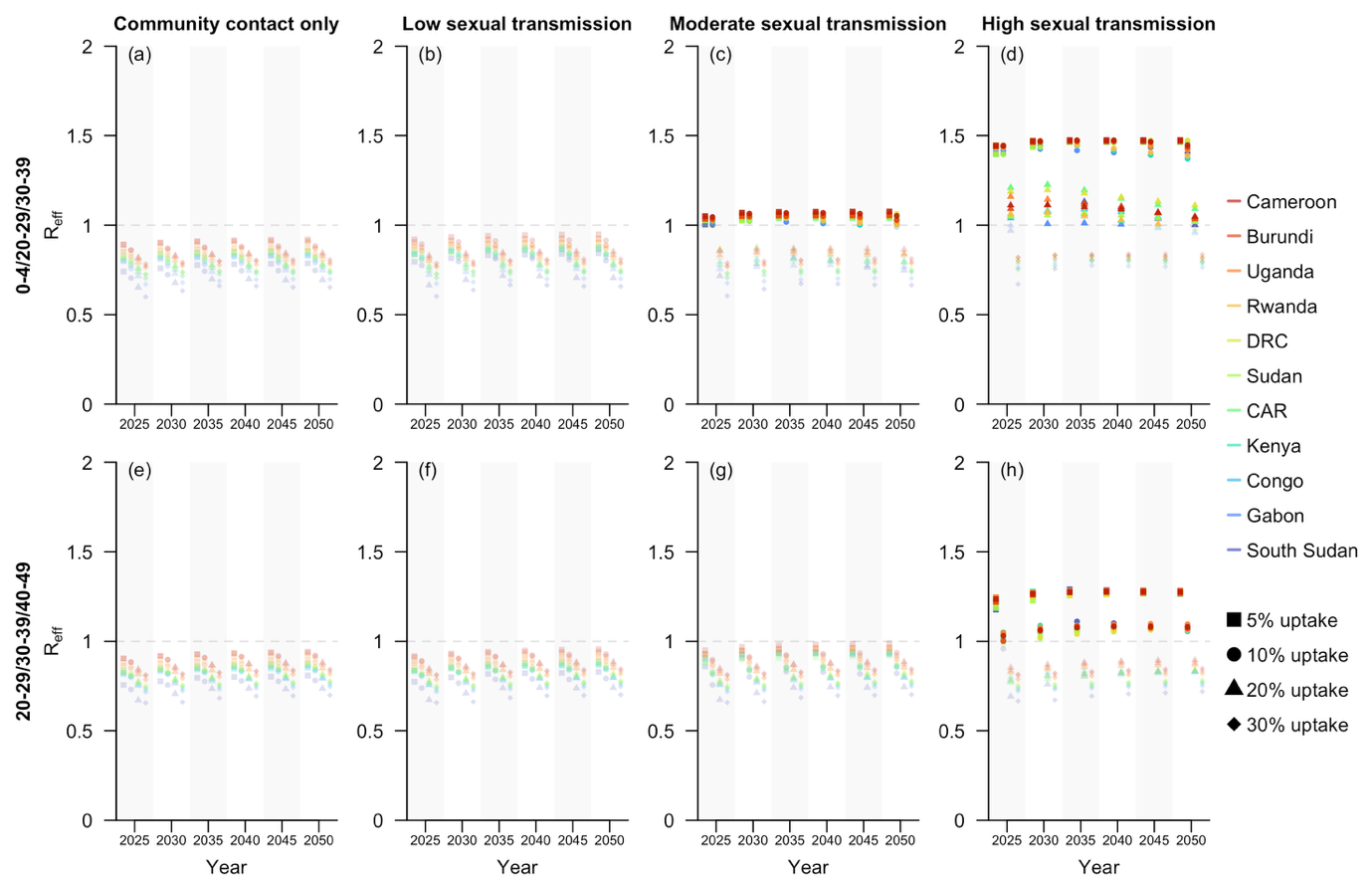


**Fig E**. Projected $R_{eff}$ at five-year intervals from 2025 to 2050 under selected vaccination strategies for sub-Saharan African countries with documented local transmission of Clade I MPXV as of December 2024 [3], based on the model calibrated using all contact components of the synthetic contact matrices [1]. These countries were ordered in descending estimated $R_{eff}$ for 2050, based on the scenario with high sexual transmission and the vaccination strategy prioritizing individuals aged 20–29, 30–39, and 40–49, sequentially (Row 2, Column 4). Four coverage rates, including 5%, 10%, 20%, and 30%, were assessed, with outcomes displayed as scattered dots across four columns within one subfigure. The rows of subfigures represent the two selected sequential vaccination strategies, while the columns correspond to the four hypothetical scenarios with varying levels of sexual transmission.

**Waning immunity from smallpox vaccines**

In the main analysis, the smallpox vaccine effectiveness was assumed to remain constant over time. To examine the potential impact of waning immunity, we conducted a sensitivity analysis by modelling the time-varying effectiveness as a non-increasing function of year $t$:

$$e_{s,t}=e_{s,2015}\left( 1-r_{e} \right)^{t-2015},$$

where $r_{e}(\geq0)$ is the annual reduction in vaccine effectiveness level. Due to the absence of individual-level vaccination timing data, we did not stratify vaccine effectiveness by age. We re-calibrated the model and estimated both $e_{s,2015}$ and $r_{e}$ using the Clade Ia outbreak data from the DRC in 2015 and 2024, while all other parameters were derived as the main analysis. The estimated vaccine effectiveness in 2015, $e_{s,2015}$, was 90.9% (95% CI: 84.4%–96.9%), while $r_{e}$ was estimated at 1.0% (95% CI: 0–2.4%), indicating an insignificant decline in effectiveness over time, which also aligns with literature [4]. Compared to the main analysis which assumed constant smallpox vaccine effectiveness, the projected $R_{eff}$s under this scenario were slightly higher, leading to increased demand for mpox vaccines (Figs F–I).


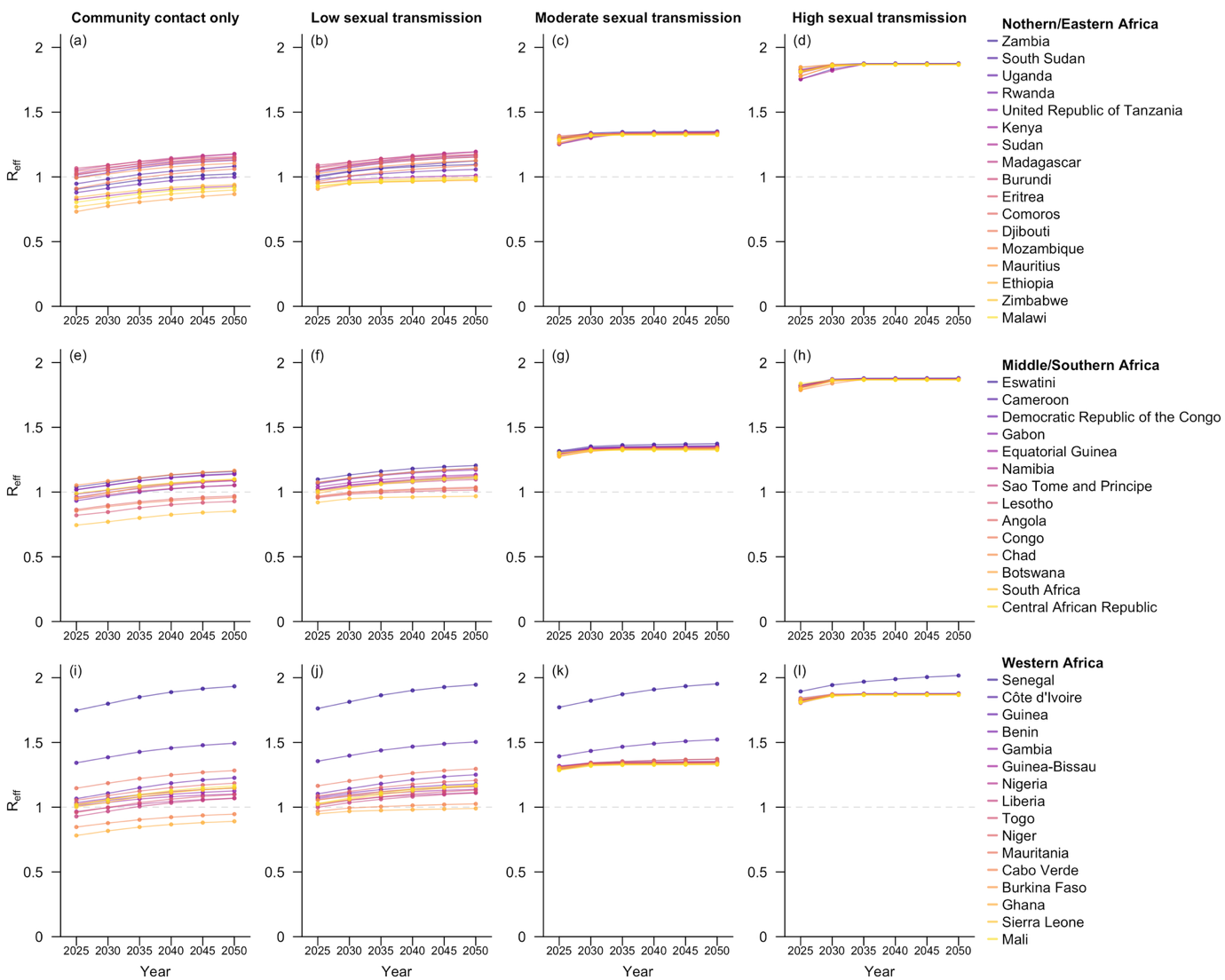


**Fig F**. Projected $R_{eff}$ for the 47 sub-Saharan African countries at five-year intervals from 2025 to 2050, adjusted for potential waning immunity from historical smallpox vaccination. Countries within the same geospatial subregions are grouped in the same row and arranged in descending order by their estimated $R_{eff}$ in 2050. The four columns correspond to the four hypothetical scenarios with varying levels of sexual transmission.

**
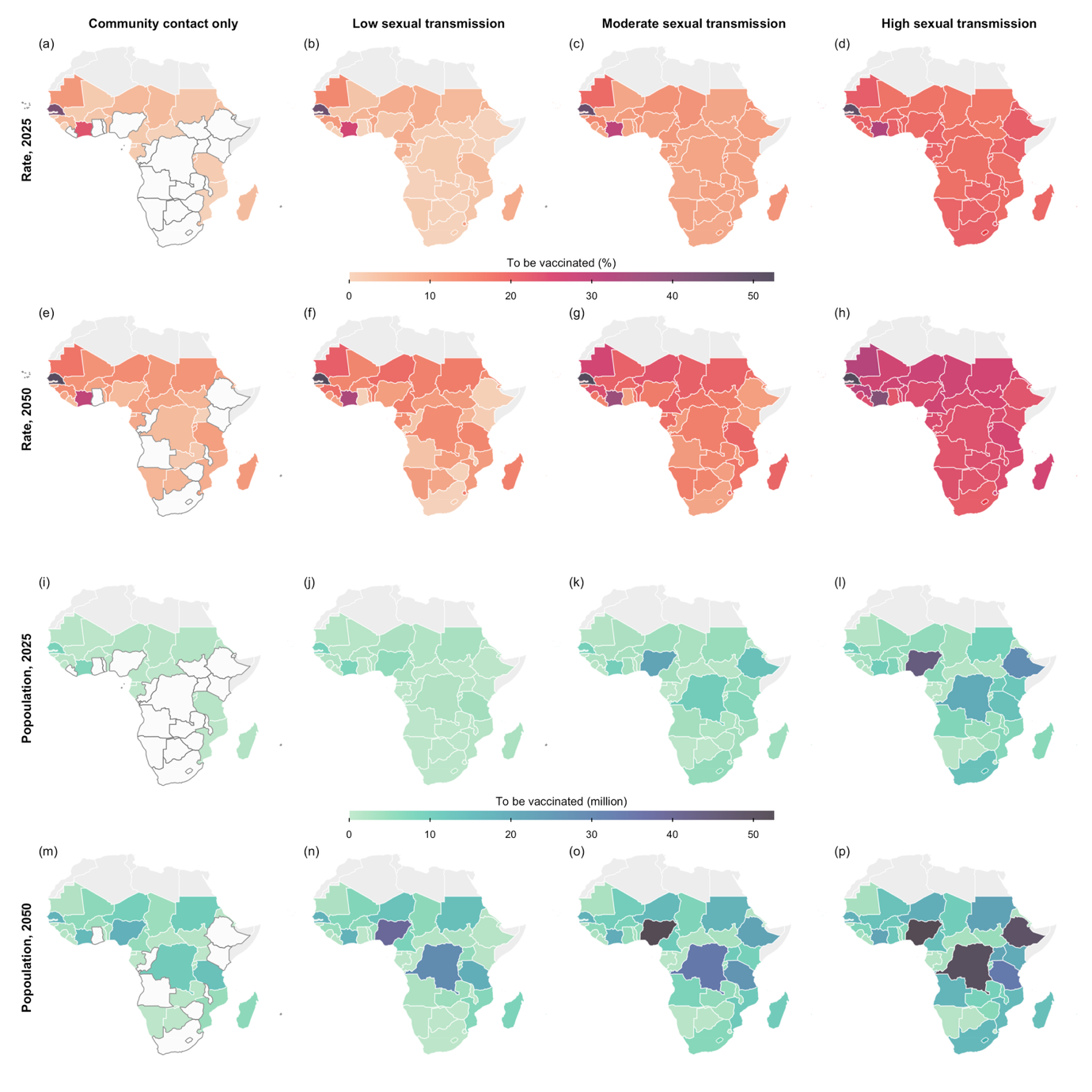
**

**Fig G.** Projected minimal vaccine demand, adjusted for potential waning immunity from historical smallpox vaccination. Shown are the vaccine coverage rates (Row 1 and 2) and the number of individuals requiring vaccination (Row 3 and 4) to prevent secondary infections in each sub-Saharan African country modelled for 2025 (Row 1 and 3) and 2050 (Row 2 and 4), assuming mass vaccination. The four columns correspond to the four hypothetical scenarios with varying levels of sexual transmission. Countries which do not require vaccination are coloured in white, with borders outlined in dark grey. The base map layer (boundaries of African countries) is sourced from Natural Earth (https://www.naturalearthdata.com), available under the Public Domain license (https://www.naturalearthdata.com/about/terms-of-use/).

**
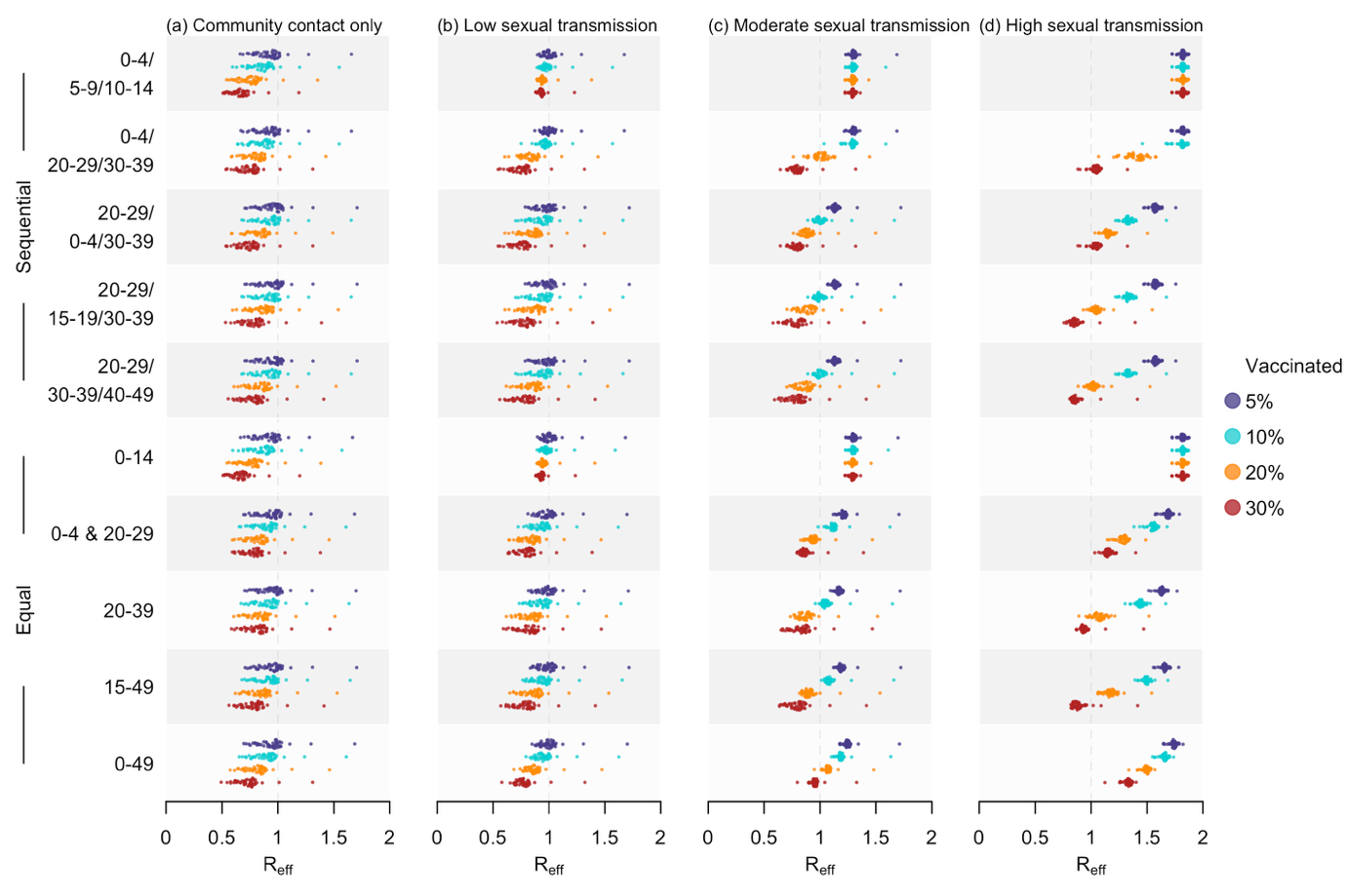
**

**Fig H**. Estimated $R_{eff}$ in 2025 under diverse vaccination strategies, adjusted for potential waning immunity from historical smallpox vaccination. Doses were allocated using two allocation methods, including sequential (first five rows) and equal (last five rows). Four coverage rates, including 5%, 10%, 20%, and 30%, were assessed, with outcomes summarised as shaded distributions in purple, blue, orange, and red, respectively. The four columns correspond to the four hypothetical scenarios with varying levels of sexual transmission.


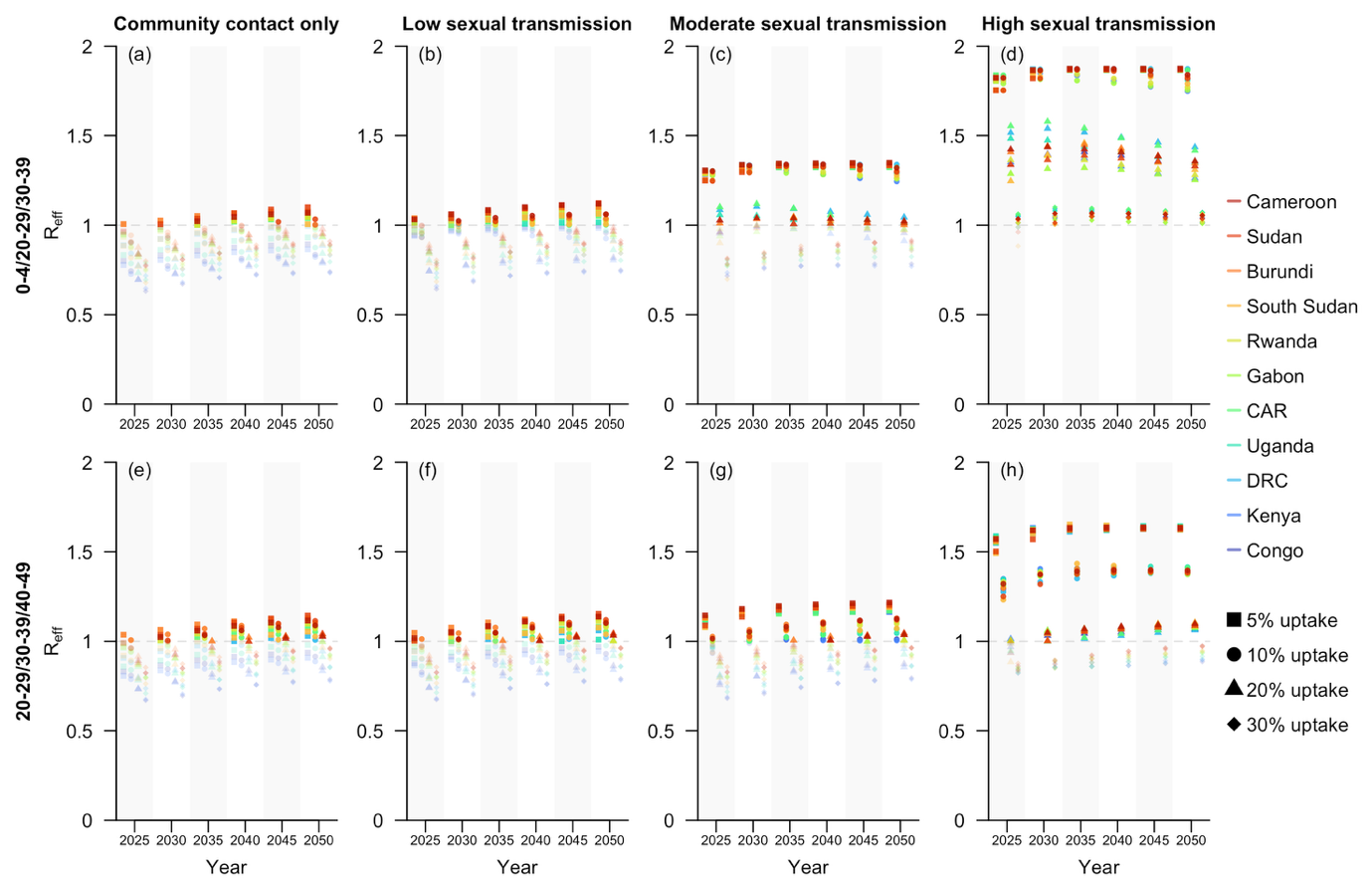


**Fig I**. Projected $R_{eff}$ at five-year intervals from 2025 to 2050 under selected vaccination strategies for sub-Saharan African countries with documented local transmission of Clade I MPXV as of December 2024 [3], adjusted for potential waning immunity from historical smallpox vaccination. These countries were ordered in descending estimated $R_{eff}$ for 2050, based on the scenario with high sexual transmission and the vaccination strategy prioritizing individuals aged 20–29, 30–39, and 40–49, sequentially (Row 2, Column 4). Four coverage rates, including 5%, 10%, 20%, and 30%, were assessed, with outcomes displayed as scattered dots across four columns within one subfigure. The rows of subfigures represent the two selected sequential vaccination strategies, while the columns correspond to the four hypothetical scenarios with varying levels of sexual transmission.

**Higher smallpox vaccine coverage in sub-Saharan African countries**

In the main analysis, the age-specific smallpox vaccine coverage was based on the mean estimates reported by Taube et al [5]. To evaluate the impact of higher-than-expected coverage on model projections, we alternatively utilised the upper bound of the 99% CI as the estimated vaccine coverage. The model was then re-calibrated, and the smallpox vaccine effectiveness ($e_{s}$) was estimated at 84.8% (95% CI: 78.5%–90.7%), lower than the estimate obtained in the main analysis. This change also introduced variation to the projected $R_{eff}$ values as well as vaccine demands in the sub-Saharan African countries (Figs J–M).


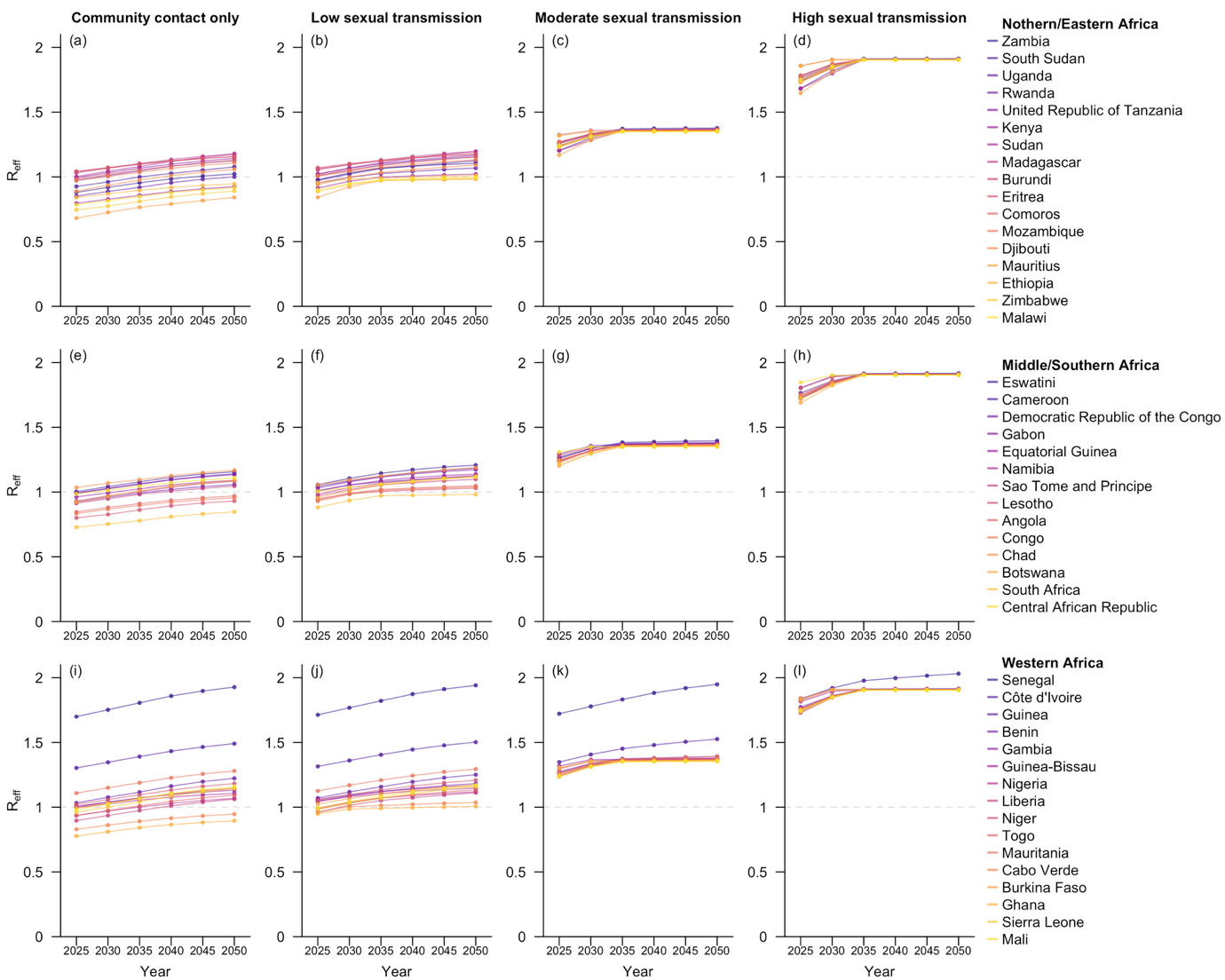


**Fig J**. Projected $R_{eff}$ for the 47 sub-Saharan African countries at five-year intervals from 2025 to 2050, assuming higher-than-expected smallpox vaccine coverage. Countries within the same geospatial subregions are grouped in the same row and arranged in descending order by their estimated $R_{eff}$ in 2050. The four columns correspond to the four hypothetical scenarios with varying levels of sexual transmission.

**
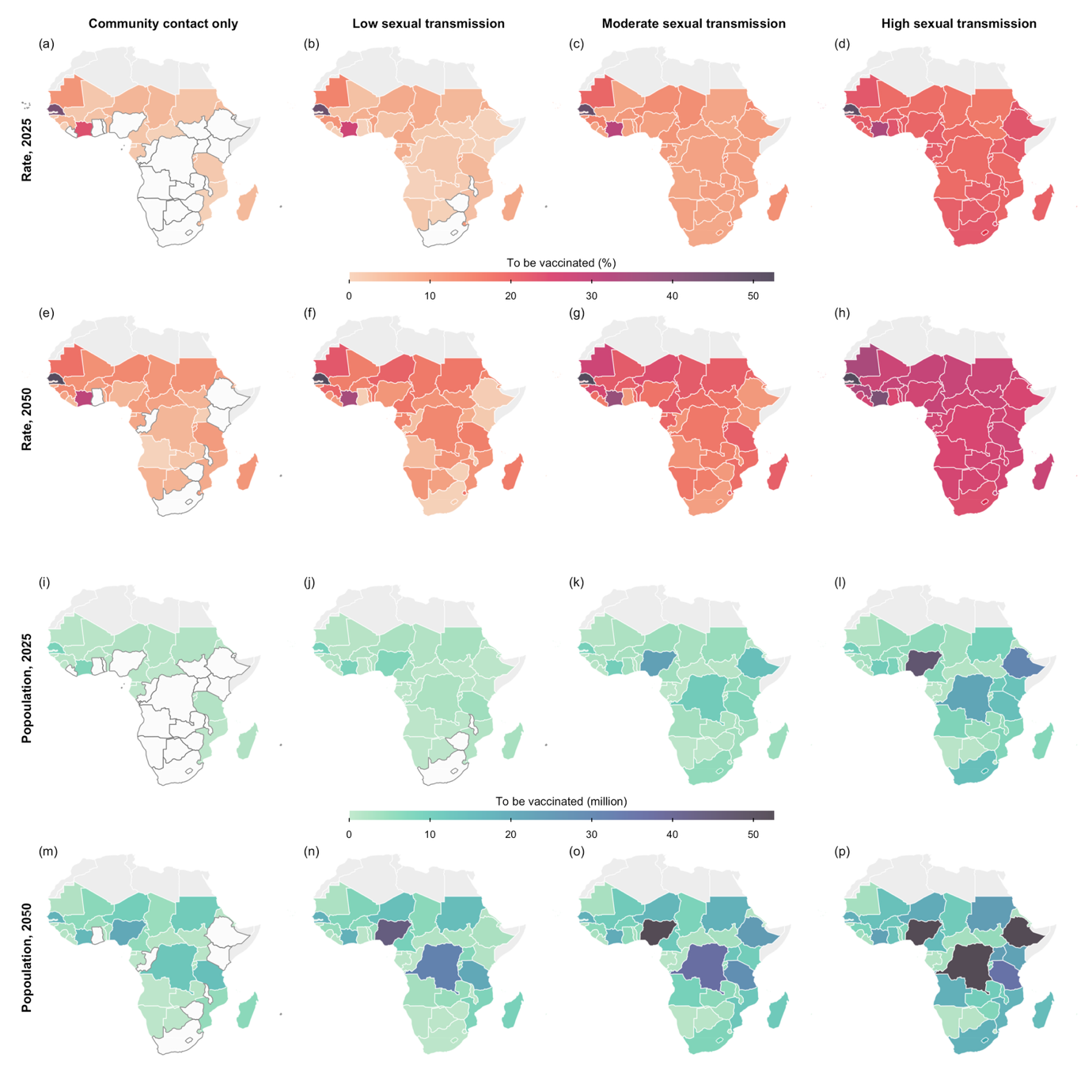
**

**Fig K.** Projected minimal vaccine demand, assuming higher-than-expected smallpox vaccine coverage. Shown are the vaccine coverage rates (Row 1 and 2) and the number of individuals requiring vaccination (Row 3 and 4) to prevent secondary infections in each sub-Saharan African country modelled for 2025 (Row 1 and 3) and 2050 (Row 2 and 4), assuming mass vaccination. The four columns correspond to the four hypothetical scenarios with varying levels of sexual transmission. Countries which do not require vaccination are coloured in white, with borders outlined in dark grey. The base map layer (boundaries of African countries) is sourced from Natural Earth (https://www.naturalearthdata.com), available under the Public Domain license (https://www.naturalearthdata.com/about/terms-of-use/).


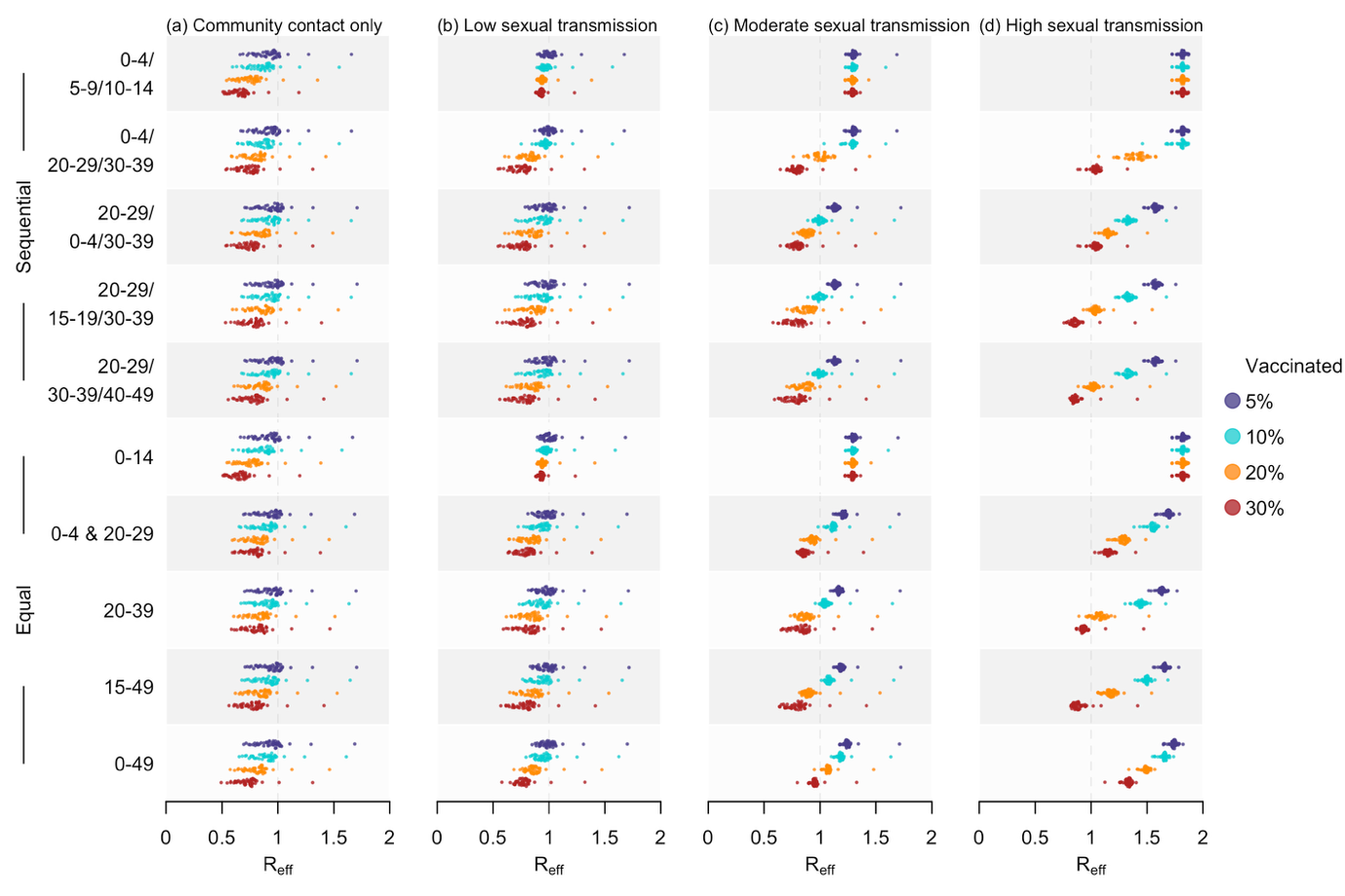


**Fig L**. Estimated $R_{eff}$ in 2025 under diverse vaccination strategies, assuming higher-than-expected smallpox vaccine coverage. Doses were allocated using two allocation methods, including sequential (first five rows) and equal (last five rows). Four coverage rates, including 5%, 10%, 20%, and 30%, were assessed, with outcomes summarised as shaded distributions in purple, blue, orange, and red, respectively. The four columns correspond to the four hypothetical scenarios with varying levels of sexual transmission.


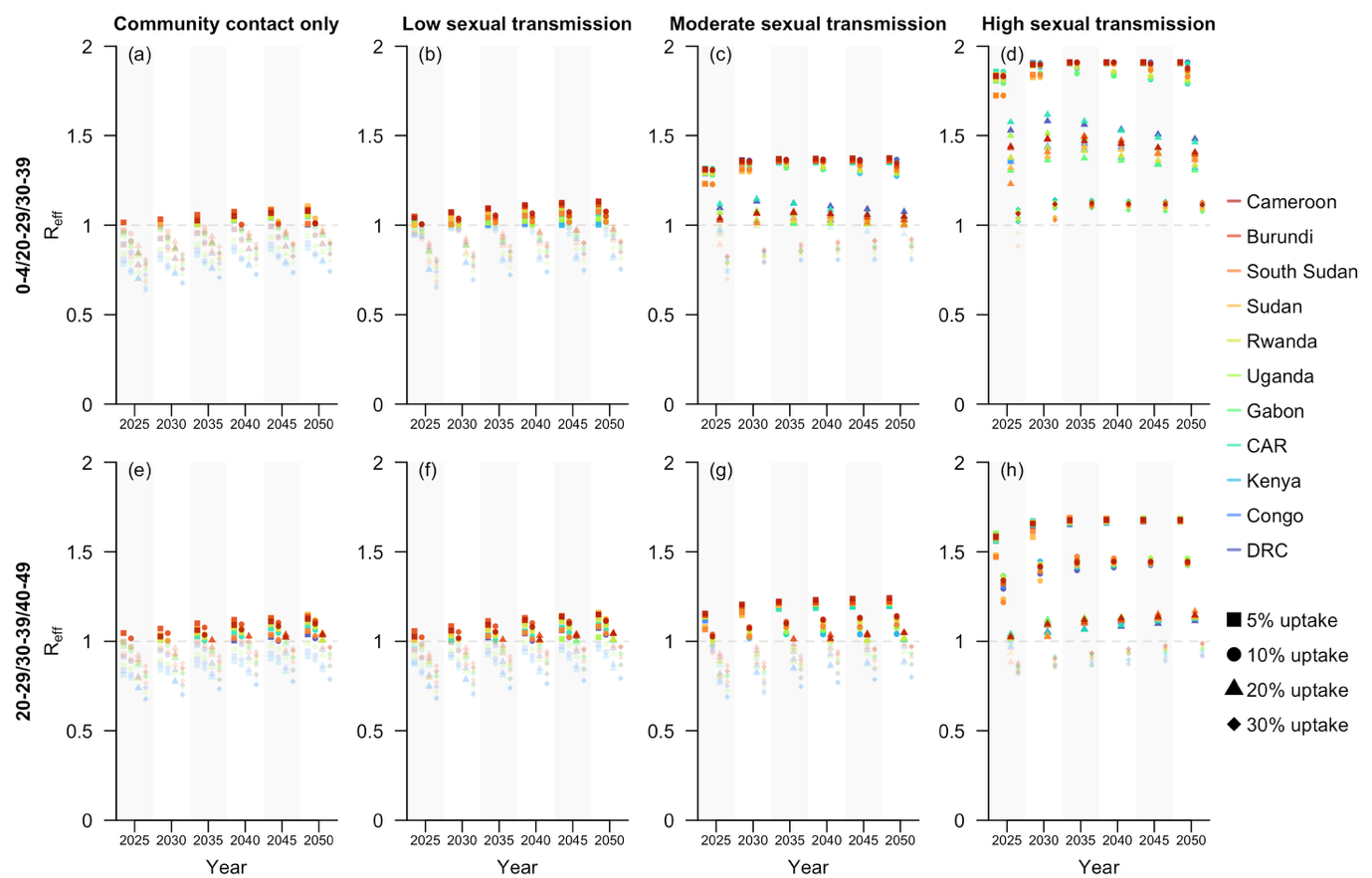


**Fig M**. Projected $R_{eff}$ at five-year intervals from 2025 to 2050 under selected vaccination strategies for sub-Saharan African countries with documented local transmission of Clade I MPXV as of December 2024 [3], assuming higher-than-expected smallpox vaccine coverage. These countries were ordered in descending estimated $R_{eff}$ for 2050, based on the scenario with high sexual transmission and the vaccination strategy prioritizing individuals aged 20–29, 30–39, and 40–49, sequentially (Row 2, Column 4). Four coverage rates, including 5%, 10%, 20%, and 30%, were assessed, with outcomes displayed as scattered dots across four columns within one subfigure. The rows of subfigures represent the two selected sequential vaccination strategies, while the columns correspond to the four hypothetical scenarios with varying levels of sexual transmission.

**Scaling the next generation matrix with uncertainty adjustments**

In the main analysis, we scaled the next generation matrix so that the estimated $R_{eff}=0.82$ for Clade Ia MPXV in 2015. For this sensitivity analysis, we introduced uncertainty by allowing $R_{eff}\sim N(0.82, {0.015}^{2})$, corresponding to a 95% CI of approximately 0.79–0.85 [6]. We then re-ran the projections of $R_{eff}$s from 2025 to 2050 and re-estimated the mpox vaccine demand across the 47 sub-Saharan African countries. The results showed only minor differences from the main analysis (Figs N–Q).


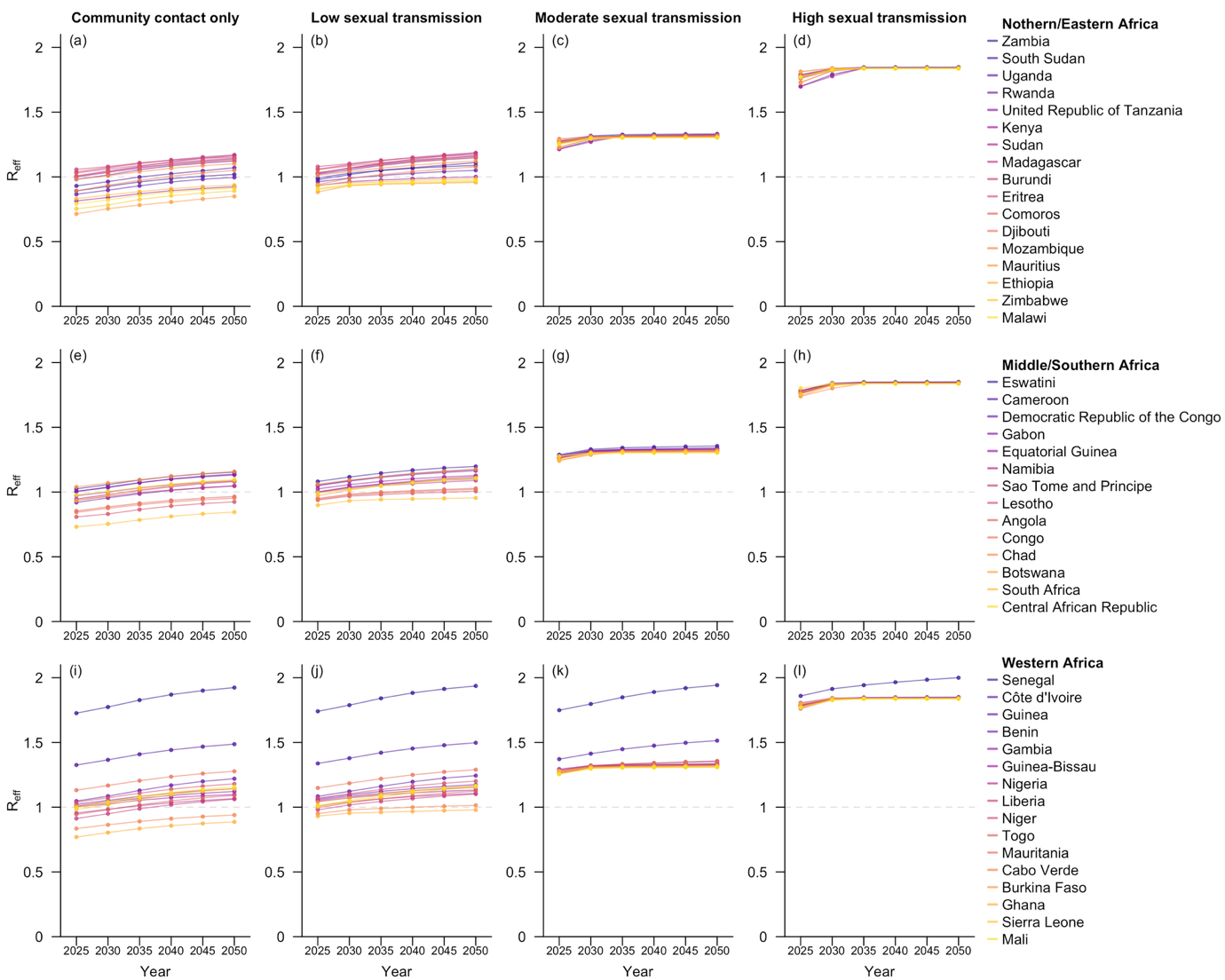


**Fig N**. Projected $R_{eff}$ for the 47 sub-Saharan African countries at five-year intervals from 2025 to 2050, with uncertainty incorporated in the scaling of the next generation matrix. Countries within the same geospatial subregions are grouped in the same row and arranged in descending order by their estimated $R_{eff}$ in 2050. The four columns correspond to the four hypothetical scenarios with varying levels of sexual transmission.

**
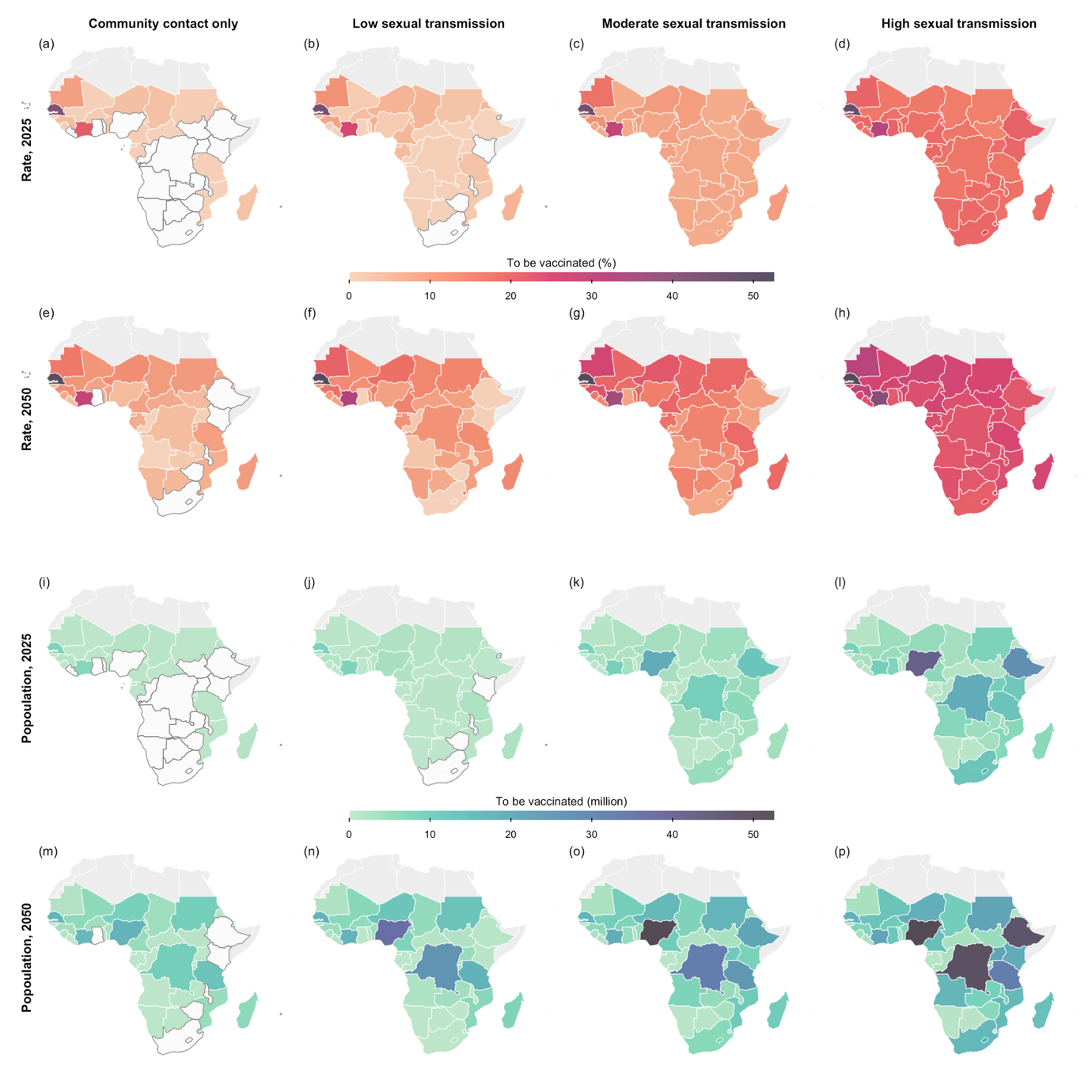
**

**Fig O.** Projected minimal vaccine demand, with uncertainty incorporated in the scaling of the next generation matrix. Shown are the vaccine coverage rates (Row 1 and 2) and the number of individuals requiring vaccination (Row 3 and 4) to prevent secondary infections in each sub-Saharan African country modelled for 2025 (Row 1 and 3) and 2050 (Row 2 and 4), assuming mass vaccination. The four columns correspond to the four hypothetical scenarios with varying levels of sexual transmission. Countries which do not require vaccination are coloured in white, with borders outlined in dark grey. The base map layer (boundaries of African countries) is sourced from Natural Earth (https://www.naturalearthdata.com), available under the Public Domain license (https://www.naturalearthdata.com/about/terms-of-use/).

**
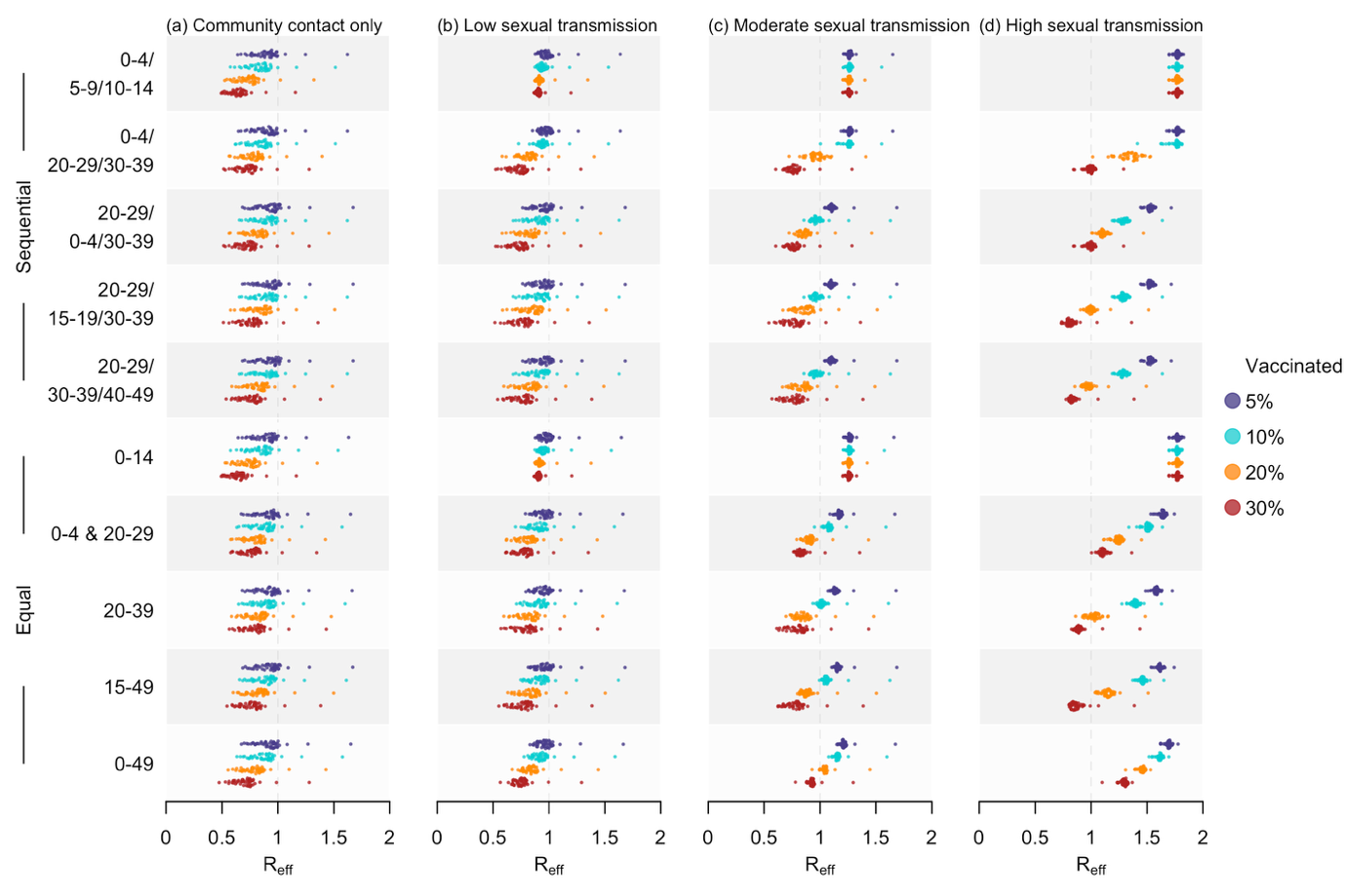
**

**Fig P**. Estimated $R_{eff}$ in 2025 under diverse vaccination strategies, with uncertainty incorporated in the scaling of the next generation matrix. Doses were allocated using two allocation methods, including sequential (first five rows) and equal (last five rows). Four coverage rates, including 5%, 10%, 20%, and 30%, were assessed, with outcomes summarised as shaded distributions in purple, blue, orange, and red, respectively. The four columns correspond to the four hypothetical scenarios with varying levels of sexual transmission.

**
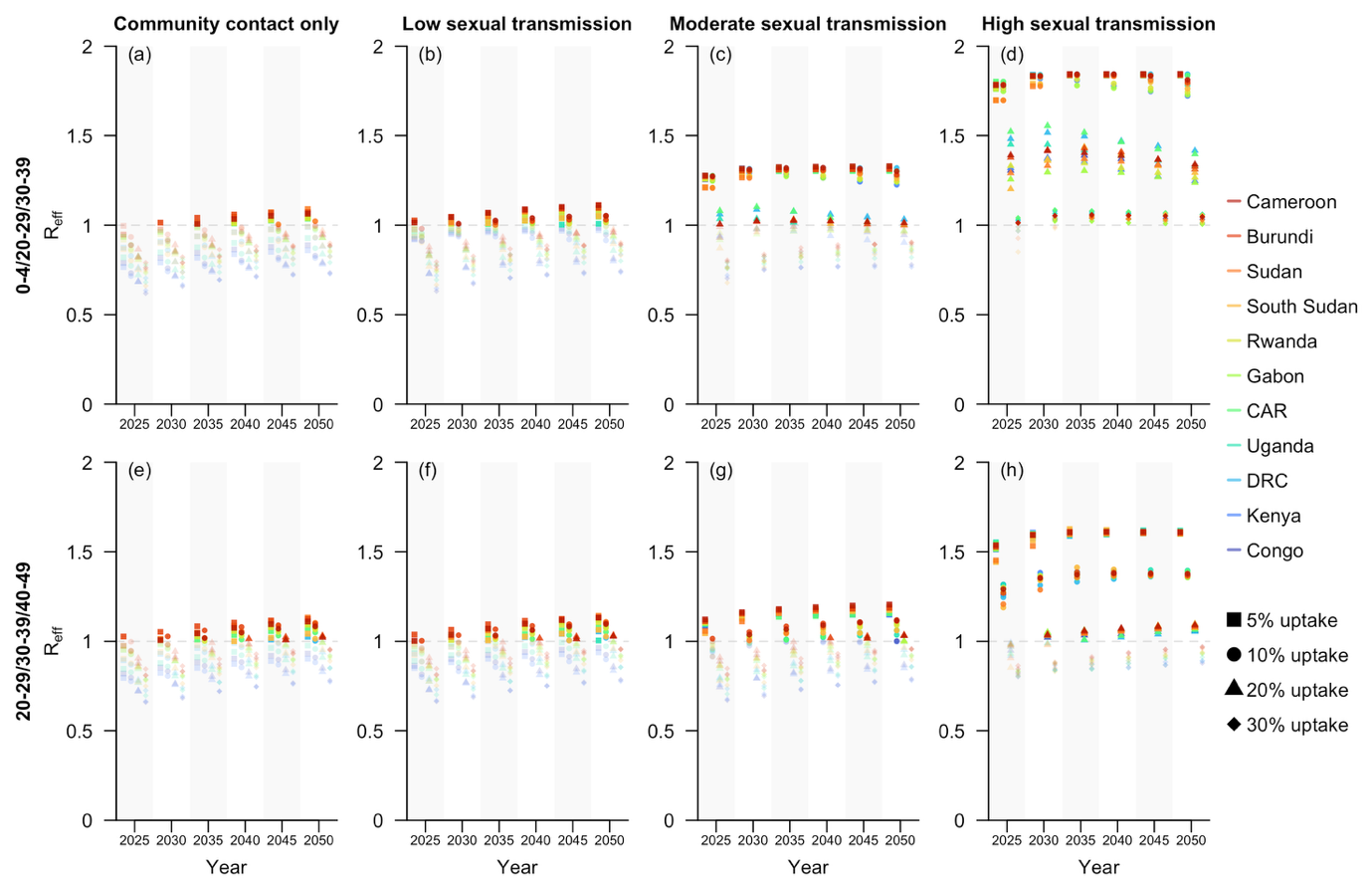
**

**Fig Q**. Projected $R_{eff}$ at five-year intervals from 2025 to 2050 under selected vaccination strategies for sub-Saharan African countries with documented local transmission of Clade I MPXV as of December 2024 [3], with uncertainty incorporated in the scaling of the next generation matrix. These countries were ordered in descending estimated $R_{eff}$ for 2050, based on the scenario with high sexual transmission and the vaccination strategy prioritizing individuals aged 20–29, 30–39, and 40–49, sequentially (Row 2, Column 4). Four coverage rates, including 5%, 10%, 20%, and 30%, were assessed, with outcomes displayed as scattered dots across four columns within one subfigure. The rows of subfigures represent the two selected sequential vaccination strategies, while the columns correspond to the four hypothetical scenarios with varying levels of sexual transmission.

**Alternative maximum uptake threshold of 70%**

We performed a sensitivity analysis to assess the impact of higher-than-expected vaccine hesitancy on mpox vaccine demand, assuming that the vaccine uptake in any age group was capped at 70% [7]. This led to a greater number of vaccine series required to achieve outbreak control and reduced effectiveness of targeted vaccination strategies with limited supply (Figs R–T).

**
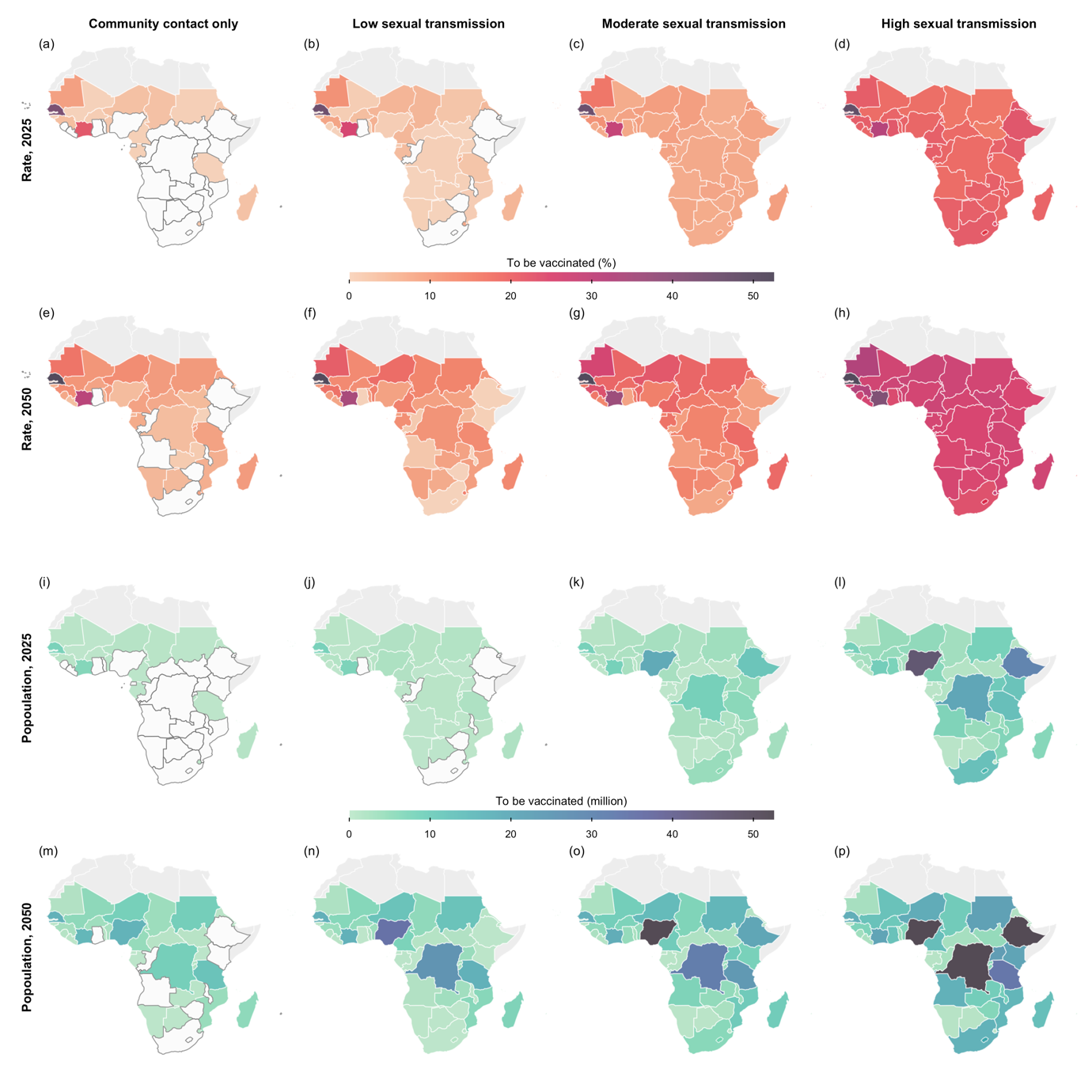
**

**Fig R.** Projected minimal vaccine demand when the maximum coverage in any group was 70% [7]. Shown are the vaccine coverage rates (Row 1 and 2) and the number of individuals requiring vaccination (Row 3 and 4) to prevent secondary infections in each sub-Saharan African country modelled for 2025 (Row 1 and 3) and 2050 (Row 2 and 4), assuming mass vaccination. The four columns correspond to the four hypothetical scenarios with varying levels of sexual transmission. Countries which do not require vaccination are coloured in white, with borders outlined in dark grey. The base map layer (boundaries of African countries) is sourced from Natural Earth (https://www.naturalearthdata.com), available under the Public Domain license (https://www.naturalearthdata.com/about/terms-of-use/).

**
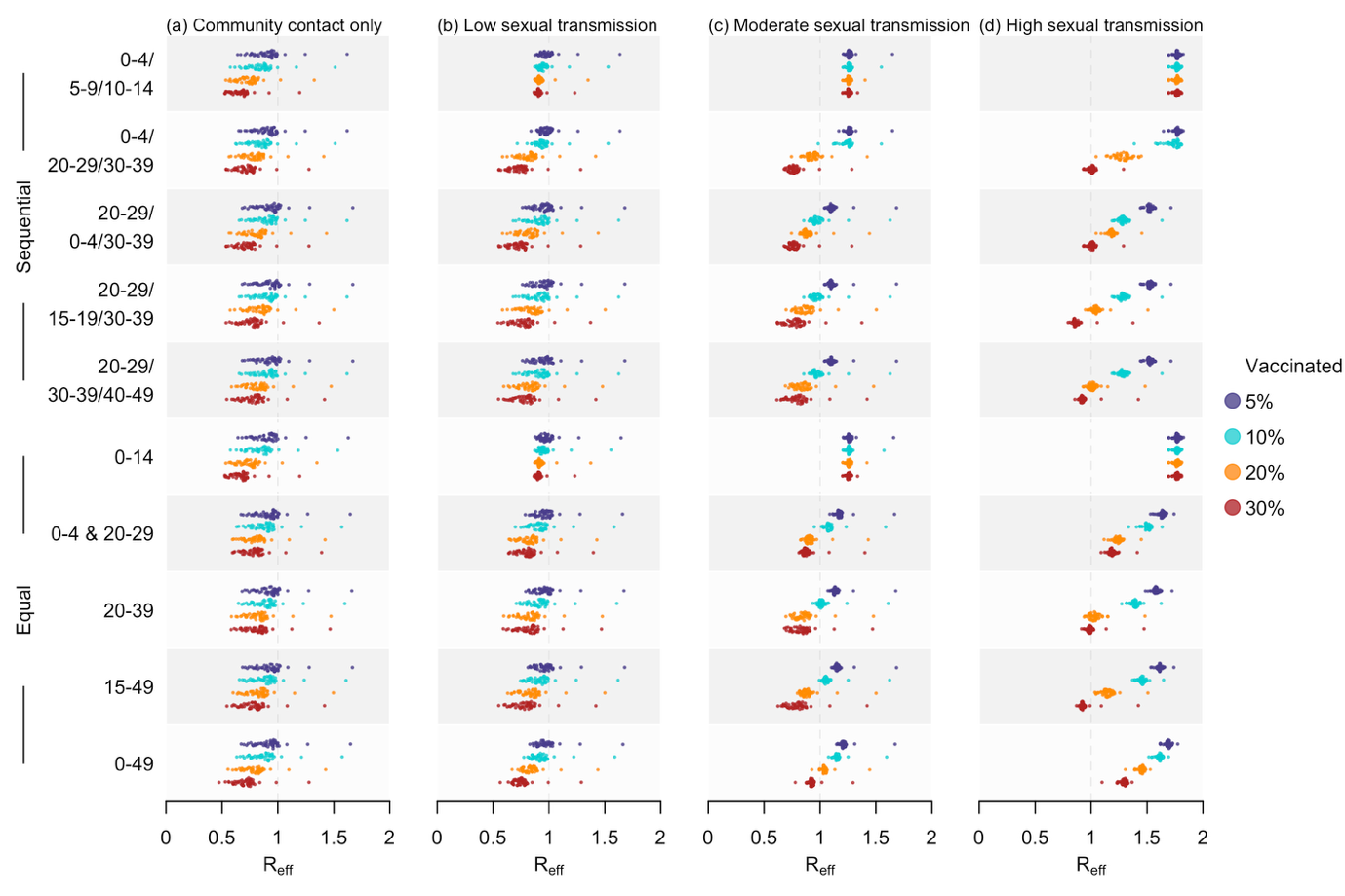
**

**Fig S**. Estimated $R_{eff}$ in 2025 under diverse vaccination strategies. Doses were allocated using two allocation methods, including sequential (first five rows) and equal (last five rows), when the maximum coverage in any group was 70% [7]. Four coverage rates, including 5%, 10%, 20%, and 30%, were assessed, with outcomes summarised as shaded distributions in purple, blue, orange, and red, respectively. The four columns correspond to the four hypothetical scenarios with varying levels of sexual transmission.

**
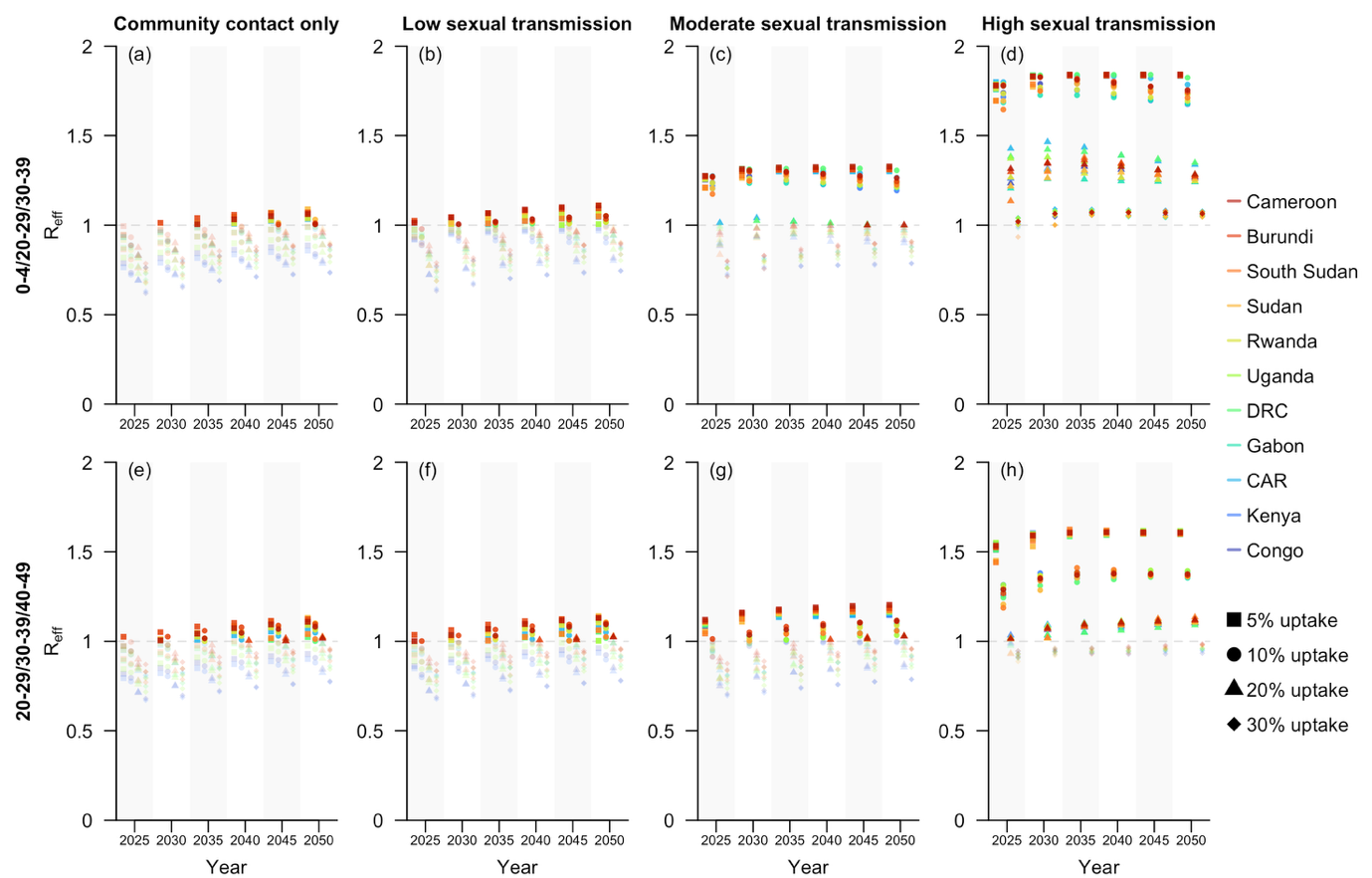
**

**Fig T**. Projected $R_{eff}$ at five-year intervals from 2025 to 2050 under selected vaccination strategies for sub-Saharan African countries with documented local transmission of Clade I MPXV as of December 2024 [3], when the maximum coverage in any group was 70% [7]. These countries were ordered in descending estimated $R_{eff}$ for 2050, based on the scenario with high sexual transmission and the vaccination strategy prioritizing individuals aged 20–29, 30–39, and 40–49, sequentially (Row 2, Column 4). Four coverage rates, including 5%, 10%, 20%, and 30%, were assessed, with outcomes displayed as scattered dots across four columns within one subfigure. The rows of subfigures represent the two selected sequential vaccination strategies, while the columns correspond to the four hypothetical scenarios with varying levels of sexual transmission.

**Alternative vaccine effectiveness of 75% or 85%**

In this sensitivity analysis, we assumed alternative mpox vaccine effectiveness of 75% or 85%. The projection results indicate that higher effectiveness is associated with lower demands for mpox vaccines, but consistent with the main analysis, both scenarios suggest to prioritise children under five years or young adults aged 20–29 for vaccination (Figs U–Z).

**
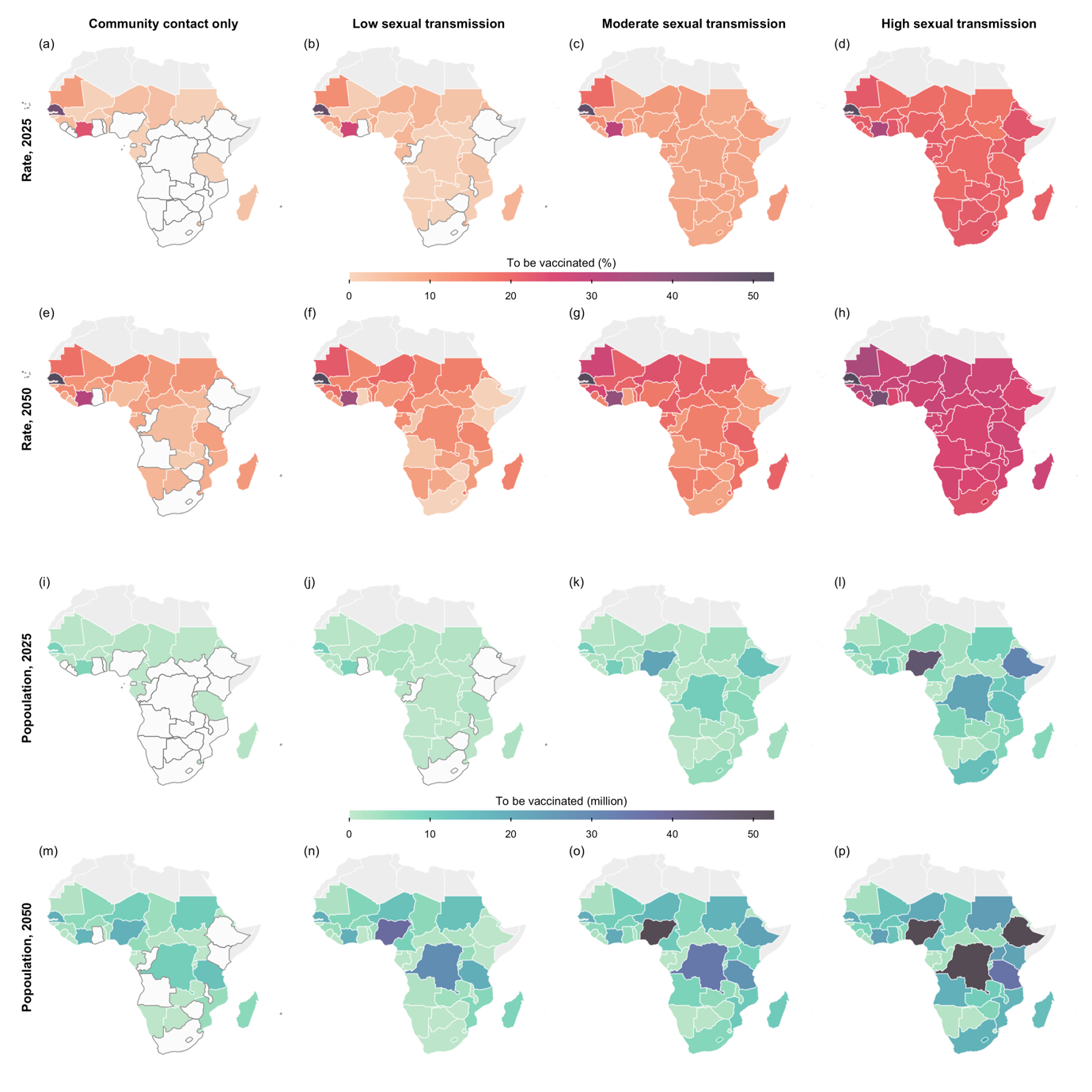
**

**Fig U.** Projected minimal vaccine demand, assuming a vaccine effectiveness of 75%. Shown are the vaccine coverage rates (Row 1 and 2) and the number of individuals requiring vaccination (Row 3 and 4) to prevent secondary infections in each sub-Saharan African country modelled for 2025 (Row 1 and 3) and 2050 (Row 2 and 4), assuming mass vaccination. The four columns correspond to the four hypothetical scenarios with varying levels of sexual transmission. Countries which do not require vaccination are coloured in white, with borders outlined in dark grey. The base map layer (boundaries of African countries) is sourced from Natural Earth (https://www.naturalearthdata.com), available under the Public Domain license (https://www.naturalearthdata.com/about/terms-of-use/).

**
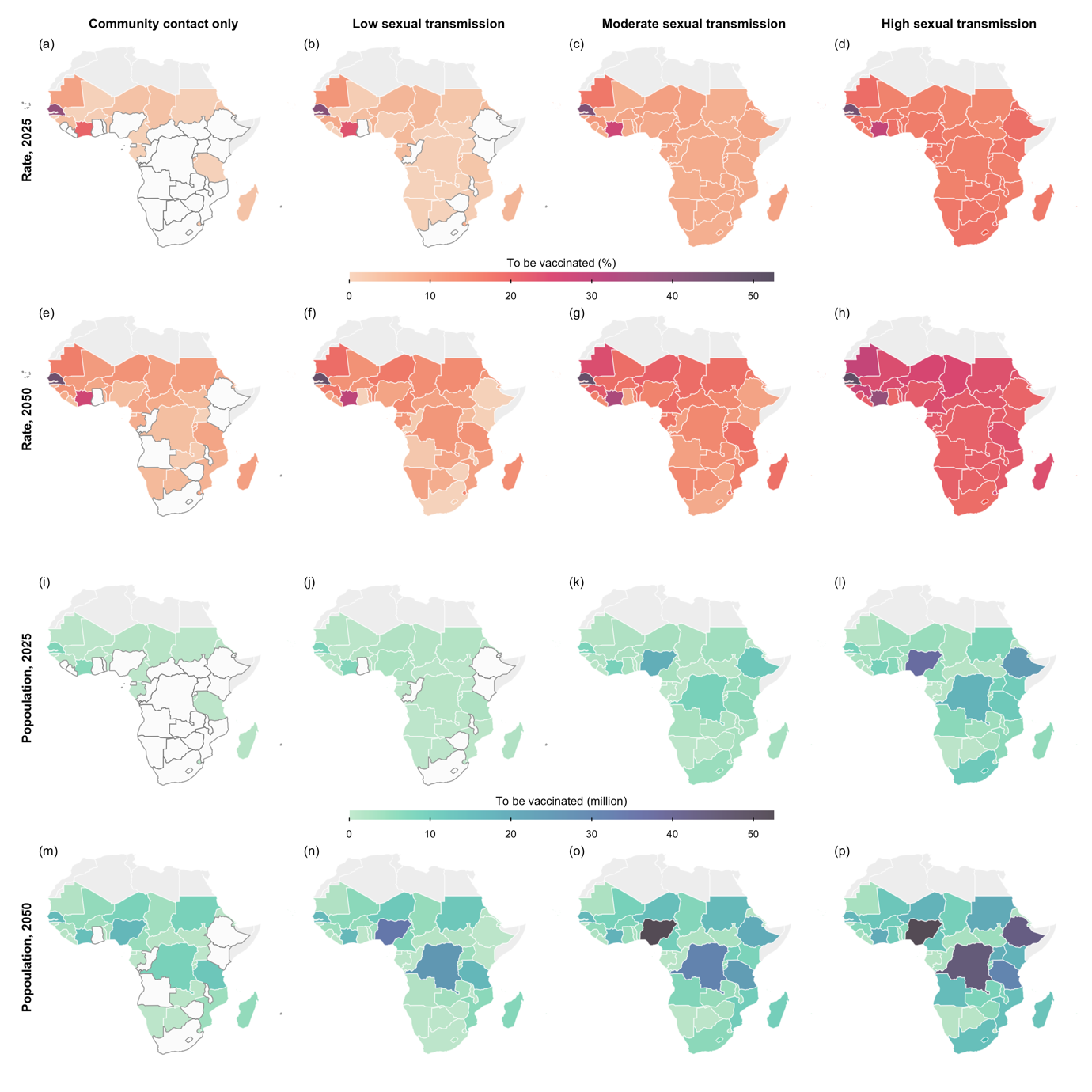
**

**Fig V.** Projected minimal vaccine demand, assuming a vaccine effectiveness of 85%. Shown are the vaccine coverage rates (Row 1 and 2) and the number of individuals requiring vaccination (Row 3 and 4) to prevent secondary infections in each sub-Saharan African country modelled for 2025 (Row 1 and 3) and 2050 (Row 2 and 4), assuming mass vaccination. The four columns correspond to the four hypothetical scenarios with varying levels of sexual transmission. Countries which do not require vaccination are coloured in white, with borders outlined in dark grey. The base map layer (boundaries of African countries) is sourced from Natural Earth (https://www.naturalearthdata.com), available under the Public Domain license (https://www.naturalearthdata.com/about/terms-of-use/).

**
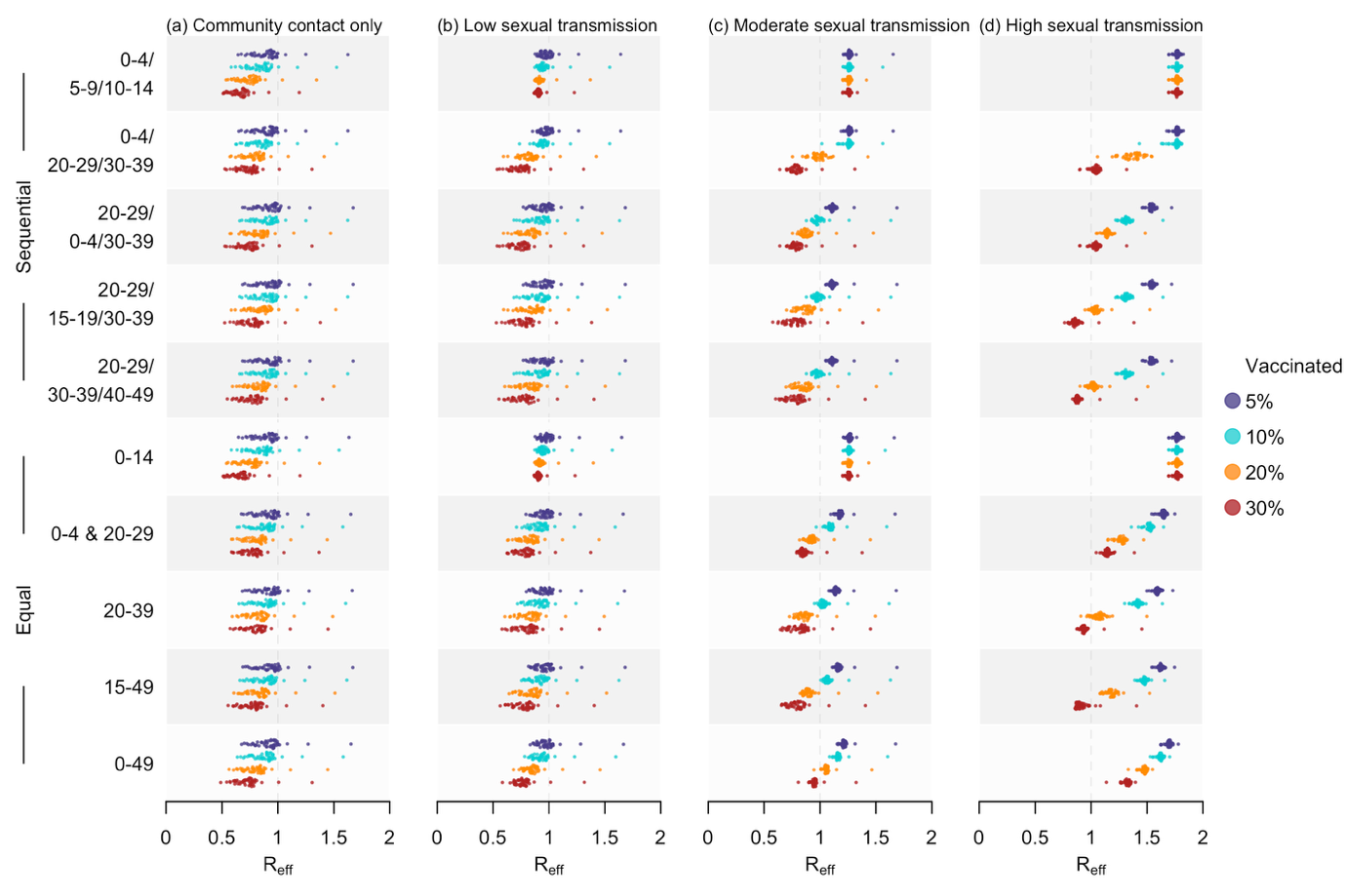
**

**Fig W**. Estimated $R_{eff}$ in 2025 under diverse vaccination strategies and a vaccine effectiveness of 75% for all age groups. Doses were allocated using two allocation methods, including sequential (first five rows) and equal (last five rows). Four coverage rates, including 5%, 10%, 20%, and 30%, were assessed, with outcomes summarised as shaded distributions in purple, blue, orange, and red, respectively. The four columns correspond to the four hypothetical scenarios with varying levels of sexual transmission.


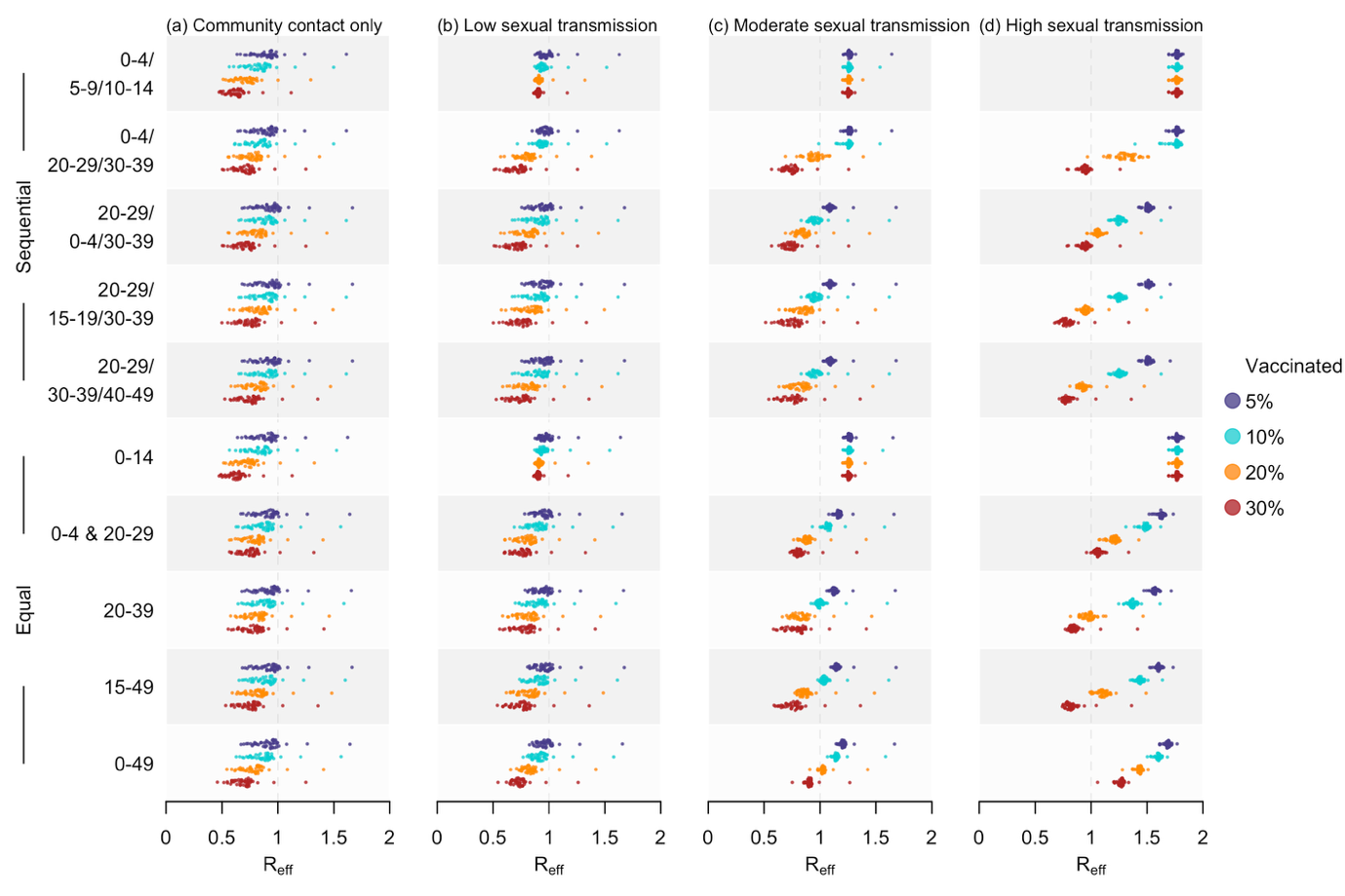


**Fig X**. Estimated $R_{eff}$ in 2025 under diverse vaccination strategies and a vaccine effectiveness of 85% for all age groups. Doses were allocated using two allocation methods, including sequential (first five rows) and equal (last five rows). Four coverage rates, including 5%, 10%, 20%, and 30%, were assessed, with outcomes summarised as shaded distributions in purple, blue, orange, and red, respectively. The four columns correspond to the four hypothetical scenarios with varying levels of sexual transmission.

**
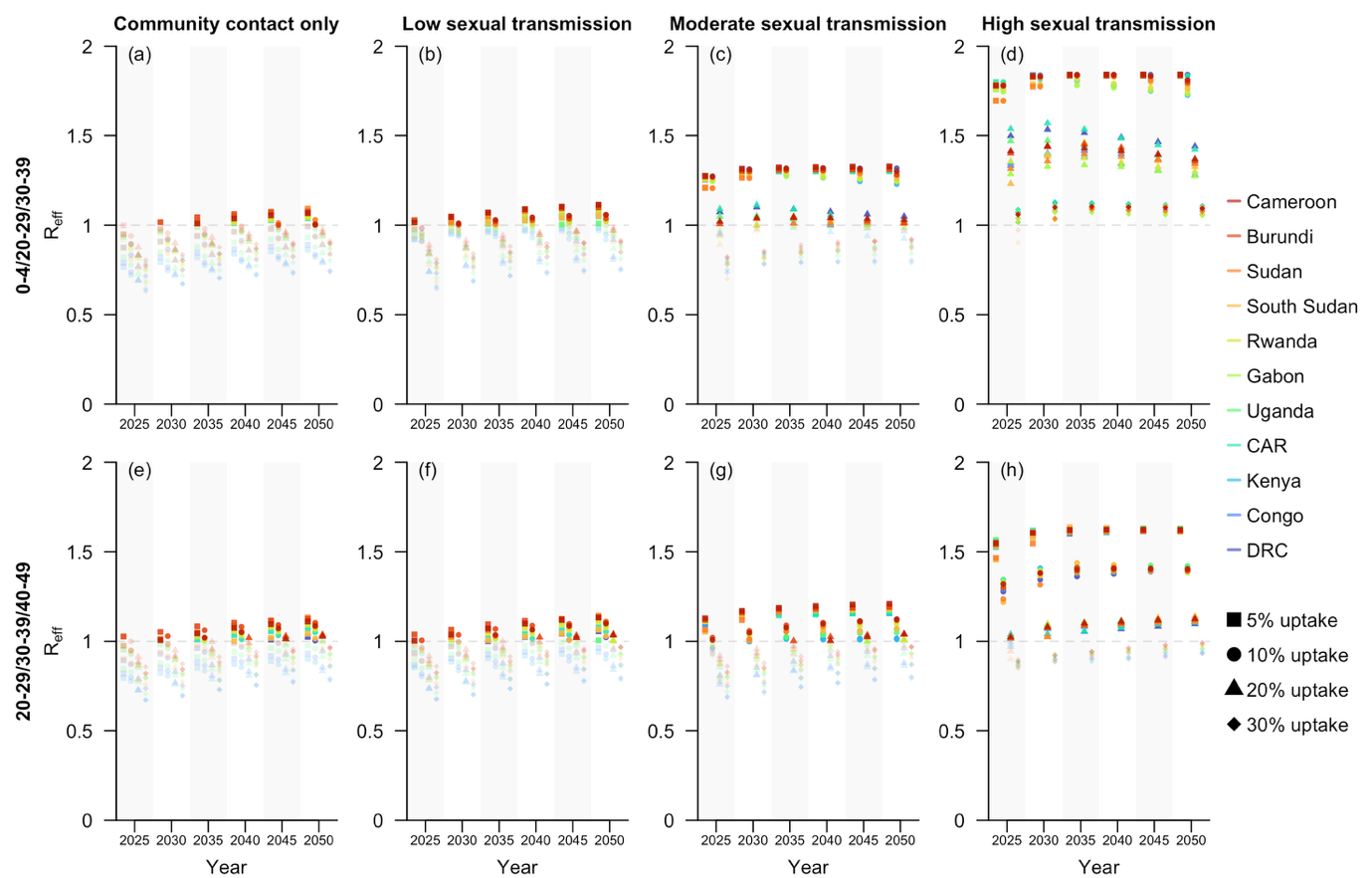
**

**Fig Y**. Projected $R_{eff}$ at five-year intervals from 2025 to 2050 under selected vaccination strategies for sub-Saharan African countries with documented local transmission of Clade I MPXV as of December 2024 [3], assuming a vaccine effectiveness of 75%. These countries were ordered in descending estimated $R_{eff}$ for 2050, based on the scenario with high sexual transmission and the vaccination strategy prioritizing individuals aged 20–29, 30–39, and 0–4, sequentially (Row 2, Column 4). Four coverage rates, including 5%, 10%, 20%, and 30%, were assessed, with outcomes displayed as scattered dots across four columns within one subfigure. The rows of subfigures represent the two selected vaccination strategies, while the columns correspond to the four hypothetical scenarios with varying levels of sexual transmission.

**
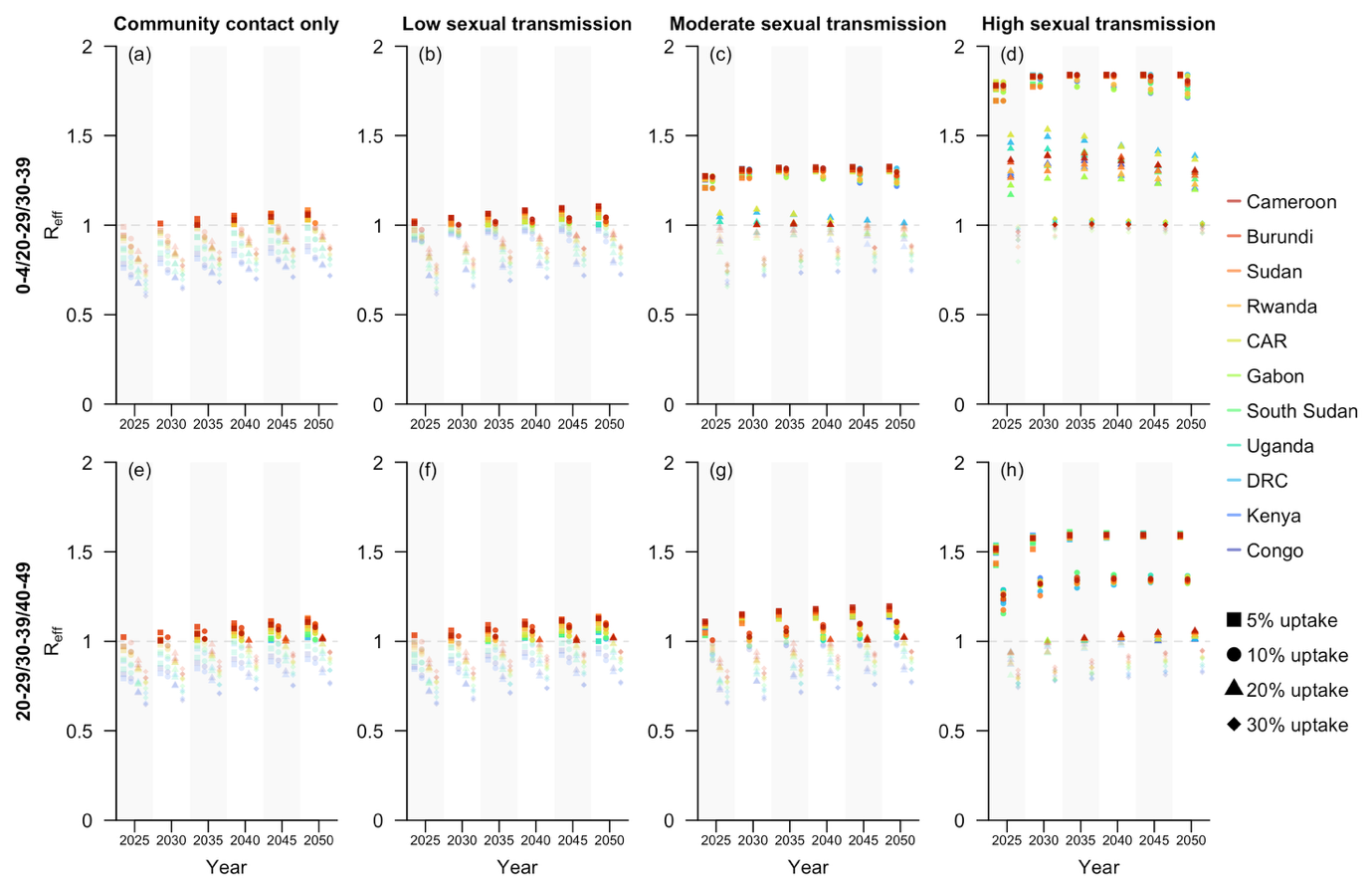
**

**Fig Z**. Projected $R_{eff}$ at five-year intervals from 2025 to 2050 under selected vaccination strategies for sub-Saharan African countries with documented local transmission of Clade I MPXV as of December 2024 [3], assuming a vaccine effectiveness of 85%. These countries were ordered in descending estimated $R_{eff}$ for 2050, based on the scenario with high sexual transmission and the vaccination strategy prioritizing individuals aged 20–29, 30–39, and 0–4, sequentially (Row 2, Column 4). Four coverage rates, including 5%, 10%, 20%, and 30%, were assessed, with outcomes displayed as scattered dots across four columns within one subfigure. The rows of subfigures represent the two selected vaccination strategies, while the columns correspond to the four hypothetical scenarios with varying levels of sexual transmission.

**References**

1. Prem K, Zandvoort K van, Klepac P, Eggo RM, Davies NG, Group C for the MM of IDC-19 W, et al. Projecting contact matrices in 177 geographical regions: An update and comparison with empirical data for the COVID-19 era. PLOS Comput Biol. 2021;17: e1009098. doi:10.1371/journal.pcbi.1009098

2. Murayama H, Asakura TR, Dickens BL, Foo JH, Jin S, Mukadi PK, et al. Roles of community and sexual contacts as drivers of clade I mpox outbreaks. medRxiv; 2024. p. 2024.10.15.24315554. doi:10.1101/2024.10.15.24315554

3. GOV.UK. Clade I mpox: affected countries. In: GOV.UK [Internet]. 2024 [cited 3 Jan 2025]. Available: https://www.gov.uk/guidance/clade-i-mpox-affected-countries

4. Eichner M. Analysis of Historical Data Suggests Long-lasting Protective Effects of Smallpox Vaccination. Am J Epidemiol. 2003;158: 717–723. doi:10.1093/aje/kwg225

5. Taube JC, Rest EC, Lloyd-Smith JO, Bansal S. The global landscape of smallpox vaccination history and implications for current and future orthopoxvirus susceptibility: a modelling study. Lancet Infect Dis. 2023;23: 454–462. doi:10.1016/S1473-3099(22)00664-8

6. Charniga K, McCollum AM, Hughes CM, Monroe B, Kabamba J, Lushima RS, et al. Updating Reproduction Number Estimates for Mpox in the Democratic Republic of Congo Using Surveillance Data. Am J Trop Med Hyg. 2024;110: 561–568. doi:10.4269/ajtmh.23-0215

7. Du M, Deng J, Yan W, Liu M, Liang W, Niu B, et al. Mpox vaccination hesitancy, previous immunisation coverage, and vaccination readiness in the African region: a multinational survey. eClinicalMedicine. 2025;80. doi:10.1016/j.eclinm.2024.103047
